# 3D point cloud data to quantitatively characterize size and shape of shrub crops

**DOI:** 10.1038/s41438-019-0123-9

**Published:** 2019-04-06

**Authors:** Yu Jiang, Changying Li, Fumiomi Takeda, Elizabeth A. Kramer, Hamid Ashrafi, Jamal Hunter

**Affiliations:** 1 School of Electrical and Computer Engineering, College of Engineering, The University of Georgia, Athens, GA 30602 USA; 2 Appalachian Fruit Research Station, United States Department of Agriculture-Agricultural Research Service, Kearneysville, WV 25430 USA; 3 Department of Agricultural and Applied Economics, College of Agricultural and Environmental Sciences, The University of Georgia, Athens, GA 30602 USA; 4 Department of Horticultural Science, North Carolina State University, Raleigh, NC 27695 USA; 5 Department of Entomology, College of Agricultural and Environmental Sciences, The University of Georgia, Athens, GA 30602 USA

**Keywords:** Plant breeding, High-throughput screening

## Abstract

Size and shape are important properties of shrub crops such as blueberries, and they can be particularly useful for evaluating bush architecture suited to mechanical harvesting. The overall goal of this study was to develop a 3D imaging approach to measure size-related traits and bush shape that are relevant to mechanical harvesting. 3D point clouds were acquired for 367 bushes from five genotype groups. Point cloud data were preprocessed to obtain clean bush points for characterizing bush architecture, including bush morphology (height, width, and volume), crown size, and shape descriptors (path curve *λ* and five shape indices). One-dimensional traits (height, width, and crown size) had high correlations (*R*^2^ = 0.88–0.95) between proposed method and manual measurements, whereas bush volume showed relatively lower correlations (*R*^2^ = 0.78–0.85). These correlations suggested that the present approach was accurate in measuring one-dimensional size traits and acceptable in estimating three-dimensional bush volume. Statistical results demonstrated that the five genotype groups were statistically different in crown size and bush shape. The differences matched with human evaluation regarding optimal bush architecture for mechanical harvesting. In particular, a visualization tool could be generated using crown size and path curve *λ*, which showed great potential of determining bush architecture suitable for mechanical harvesting quickly. Therefore, the processing pipeline of 3D point cloud data presented in this study is an effective tool for blueberry breeding programs (in particular for mechanical harvesting) and farm management.

## Crop breeding: 3D measurement of blueberry bush shape

Researchers in the United States have developed a 3D imaging technique to quantitatively measure the shape of blueberry bushes in order to evaluate their suitability for mechanical harvesting. The team, led by Changying Li of the University of Georgia, used handheld LiDAR scanners to collect 3D data from bushes in blueberry fields. Their analysis pipeline converted these data into a description of the bushes, including height, width, volume, crown size, and parameters describing the shape. While some traits matched manual measurements better than others, the analysis described bush shape sufficiently to distinguish varieties. The team also created a tool to visualize key parameters related to suitability for mechanical harvesting. This study provides a promising tool to evaluate and manage varieties of blueberries and similar crops, though further work is needed to speed up data collection.

## Introduction

Blueberries are nutritious fruit, containing ample amounts of phytochemicals (e.g., antioxidants) beneficial to human health^1^. The United States (US) is the largest blueberry producer and consumer in the world, and the recognition of blueberry economic and nutritional values have prompted cultivation of blueberries in other countries (e.g., Chile, China, Mexico, Peru, Australia, and European countries)^2^. Thus, an increasing demand for blueberries has been foreseen, requiring improvements in blueberry production technology and fruit quality in the future. These improvements require breeding programs to develop superior genotypes that are better adapted to different climates and modern agriculture production practices, including fruit harvesting with over-the-row (OTR) mechanical harvesters. Phenotyping technologies provide various traits for genotype evaluation in breeding programs^3–5^. These traits can also be used for management decision-making in commercial production fields such as the ability to use mechanical harvesting methods with limited (or even no) impacts on fruit quality.

Bush architecture is important for tree/shrub crops, because it usually can be used for growth evaluation, biomass estimation, yield prediction, harvest efficiency improvement, and utilization of plant protection products such as pesticides^6^. Size and shape are two important aspects of bush architecture. Size-related traits indicate the overall growth status of bushes, which are related to yield potential. Studies were conducted to use size-related traits to evaluate blueberry vegetative growth under various environments, showing a reasonable correlation between those traits (e.g., bush height) and berry yield^7,8^. Bush shape describes the geometry of bushes, which is an important factor affecting the performance of OTR mechanical harvesters. Some blueberry growers are already using OTR mechanical harvesters. More growers expect to rely on OTR mechanical harvesters to pick blueberries for fresh market, addressing the challenges of increasing harvest labor cost and anticipated insufficient labor force. To maximize the performance of OTR mechanical harvesters, blueberry plants ideally should have a narrow and small crown (e.g., small cross-section area near the ground) and a vase-shaped canopy^9–11^. A narrow and small crown is easy to tighten with catch plates of OTR harvesters, leaving small gaps between the bush and the catch plates to prevent berries from falling to the ground^12,13^. This reduces yield losses due to mechanical harvesting. A vase-shaped canopy positions fruit away from the central crown of a blueberry plant, providing a relatively open area for berries to drop onto harvester catch plates. This reduces external impacts (and thus potential bruises) on machine-harvested berries for better fruit quality and longer shelf-life. To date, the determinations of bush dimension, crown size, and bush shape have largely relied on manual assessments, which are subjective and laborious.

Crop size-related traits have been widely studied using two-dimensional (2D) and three-dimensional (3D) imaging modalities. 2D imaging approaches were primarily used to extract unitless ratios or traits in the unit of image pixel^14^. When a reference object is provided or the imaging system is pre-calibrated, extracted traits can be converted to real world units. Conversion models are usually established for greenhouse- or chamber-based phenotyping systems due to the easy deployment of reference objects and precise configuration of a pre-calibrated imaging system^15–18^. When the distance between the canopy and camera is relatively consistent, unitless ratios (e.g., canopy coverage ratio) are comparable over different data collection periods and thus they have been extracted in many 2D imaging-based studies^14^. With the increased availability of 3D sensing approaches, researchers are starting to frequently use 3D imaging techniques for measuring size-related traits^14,19^. Previous studies intensively investigated size-related traits at the plant and canopy levels for tree and shrub crops such as apples^20^, pears^20^, grapes^20–22^, hickories^23^, olives^24^, almonds^25^, peaches^26^, and blueberries^27^. These studies showed a general trend that the accuracy of crop size measurement mostly depended on point cloud quality, which is determined by sensing range and imaging approaches. Photogrammetry-based 3D imaging approaches (e.g., the structure from motion (SfM)) are inexpensive and can provide detailed point cloud data, but they require considerable computational resources for 3D reconstruction. The quality of reconstructed point clouds is significantly affected by ambient conditions such as illumination changes and wind. In addition, the SfM technique requires the use of reference objects to scale reconstructed point clouds, if no metric data (e.g., accurate metric positions of image acquisition) are provided. In such cases, reference targets need to be included in the imaging scene, introducing potential challenges for large field experiments (e.g., over several hundreds of plots)^28–30^. Active 3D imaging instruments (e.g., LiDARs) are costly but usually provide fast 3D measurements. Some active instruments have particular outdoor configurations (e.g., special emitting illumination sources) to dramatically improve the accuracy and repeatability of 3D reconstruction in the field. However, occlusions can lead to incomplete scanning of objects, presenting difficulties in trait measurement. For instance, it would be difficult to measure plant organs and branches under the canopy because they cannot be imaged by instruments using a single sensing angle. Recently, a handheld mobile laser scanner was developed so that full-view point clouds can be obtained^31^. Two studies demonstrated that the handheld laser scanner could obtain point clouds with much less missing points due to occlusion for forest structure characterization and inventory^32,33^. Thus, it is worthwhile to explore the use of this laser scanner to obtain point cloud data for measuring size-related traits of shrub crops, especially the traits of plant parts under the canopy such as crown size of blueberry plants.

Shape analysis methods can be grouped into two categories: descriptive methods and outline-based methods^34^. Both methods have been commonly used to analyze shapes of fruits, vegetables, and plant leaves. Descriptive methods usually define landmark points that can be used to derive ratios, angles, and their combinations for quantifying object shapes. Descriptive methods have been used to study the shape of tomatoes^35,36^, eggplants^37^, vineyard grape leaves^38^, and peppers^39^. Outline-based methods rely on advanced mathematical tools (e.g., curve functions and elliptical Fourier analysis (EFA)) to quantitatively describe object shapes using transformed features. Studies reported the use of EFA for analyzing the shape of mistletoe berries^40^, cotton leaves^41^, oranges^42^, ash tree fruit^43^, and persimmons^44^. Shape descriptors defined in descriptive methods have clear physical meanings, which can be easily interpreted and compared. However, defining descriptors requires a good understanding of domain needs and knowledge, involving extra efforts from domain experts. In contrast, features extracted using outline-based methods usually have no direct physical meaning, which requires visualization tools for feature interpretation and understanding. Outline-based methods use general mathematical models/framework to calculate shape features, which require almost no domain knowledge for conducting data analyses. It is also possible to use both methods for a comprehensive analysis because shape descriptors from the two methods could be complementary to each other^45^. In fact, both methods would be suitable for bush shape analysis for two reasons: (1) there is a clear physical definition of optimal bush shape for mechanical harvesting, and thus it would be straightforward to define landmark points to extract shape features; (2) previous studies^40,46^ demonstrated that a curve function (path curve) can effectively depict differences between vase shape, cone shape, and round shape, which would be worthwhile to explore.

To the best of our knowledge, only one study from our group has reported on the potential of using 3D imaging to extract size-related traits and shape descriptors of bush crops such as blueberries^27^. The study used an unmanned aerial system to acquire oblique images of blueberry bushes from approximately 3 m above the ground for reconstructing point clouds using the SfM technique. It achieved a strong correlation (*R*^2^ = 0.92) between imaging and manual measurements of bush dimensions (e.g., height and width) with an root mean square error (RMSE) of 0.1 m, indicating a high system measurement accuracy. However, correlations were less desirable (*R*^2^ = 0.38–0.55) between imaging and manual measurements for crown size, which is one of the most important parameters of machine harvest efficiency. The undesirable correlations occurred primarily due to the limitation of data collection system. When images were acquired on top of bushes regardless of using nadir or oblique perspectives, bush canopy occluded plant architecture close to the ground, leading to an incomplete 3D reconstruction of bush crown and thus inaccurate measurement of crown size. In addition, some shape descriptors defined in the study may not have been effective in identifying desired bush shapes. For instance, “blockiness” was defined as the ratio of widths at 85% and 65% canopy heights, but it had no relation to the position of the widest canopy cross section, which is the determinant among round (the widest cross section in the middle), conical (the widest cross section in lower canopy), and vase shapes (the widest cross section in upper canopy). In fact, bush canopy can be round, conical, and vase-shaped for the same “blockiness” value. Therefore, it is necessary to address aforementioned issues and provide improved approaches in both data collection and analysis for measuring size-related traits and bush shape. These approaches would be particularly useful for breeding programs to select blueberry genotypes suited to machine harvesting.

The overall goal of this study was to develop a 3D imaging approach to measure blueberry bush dimensions and shape in the field. Specific objectives were to: (1) evaluate the accuracy of sensor measurements for objects with standard shapes in field conditions; (2) develop data processing algorithms to extract size-related traits (bush dimensions and crown size) and shape descriptors of blueberry bushes; (3) evaluate the accuracy of proposed method; and (4) explore the usefulness of bush shape descriptors for machine harvesting and farm management.

## Results

### Reconstructed point cloud data

**Fig. 1:**
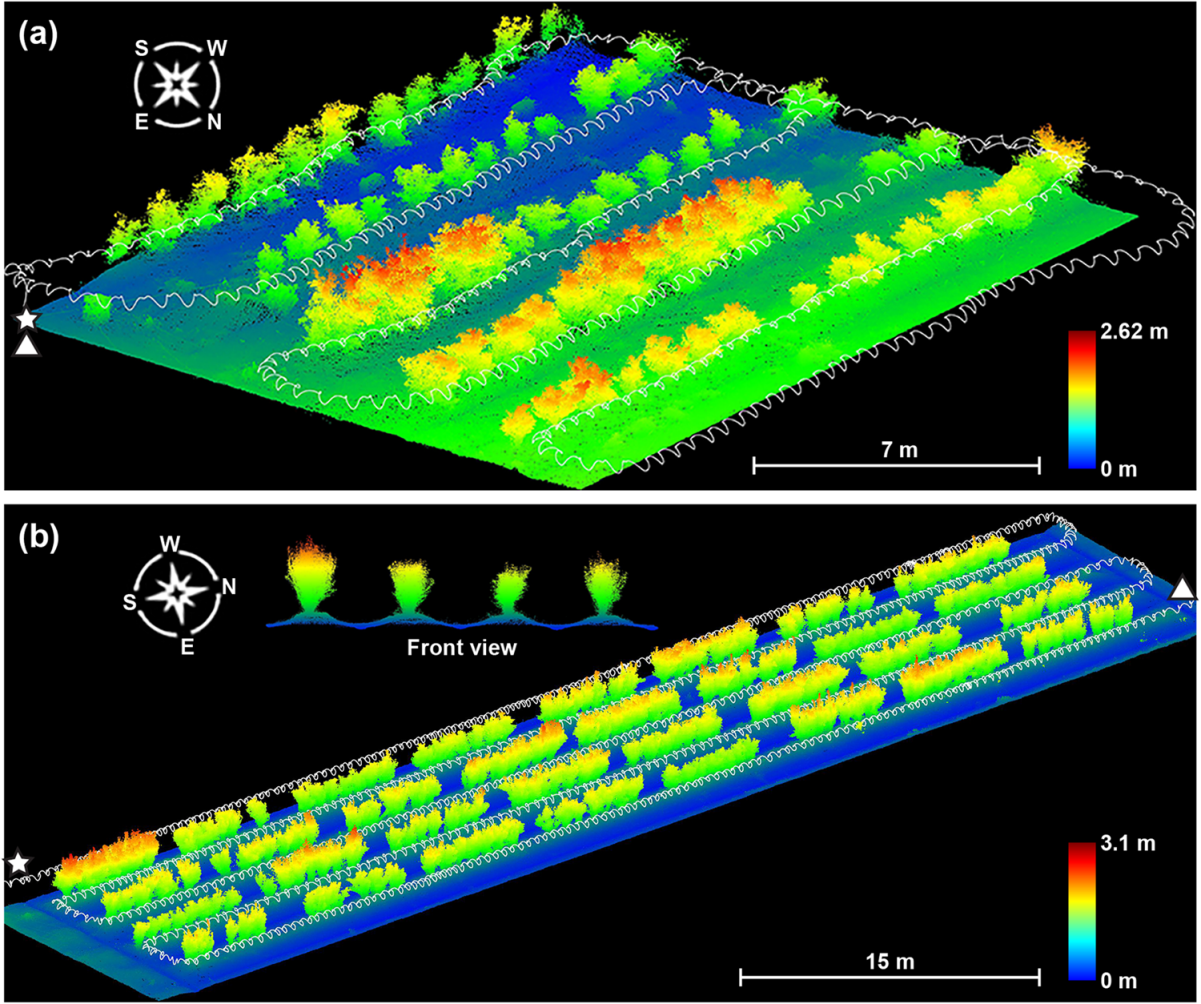
Point cloud data collected using a handheld mobile laser scanner in two fields. The point cloud of blueberry field **a** Horticulture Farm of the University of Georgia and **b** Horticulture Research Station in North Carolina. The displayed point clouds were cropped to remove irrelevant objects. White lines indicate the walking path and sensor node oscillations of data collection sessions, and star and triangle markers show the starting and ending points of the walking path

Reconstructed results contained a scanning trajectory and raw point clouds for scanned areas (Fig. 1). In trajectories, the lowest position indicated the starting and ending points of each data collection session, and starting and ending points could be further differentiated based on the relative movement direction. The starting point was the origin in each scanned point cloud. The waveform of trajectories reflected the oscillation of the LiDAR node.

Raw point clouds were rendered by color using point height information, with blue to red representing low to high values. The blueberry field at the Horticulture Farm of the University of Georgia showed an obvious sloped terrain: the southern side (top part in the figure) was at a lower elevation than the northern side (bottom part in the figure), resulting in different height values (colors) of ground points (Fig. 1a). In contrast, the terrain elevation was relatively level (ground points looked in similar blue colors) in the field at the Horticulture Research Station in North Carolina, but clear color contrasts were observed between furrows (dark blue) and plant beds (turquoise). These differences increased data variability and could be particularly challenging for point cloud preprocessing, but algorithms developed in this study successfully removed ground and noise points, suggesting its generalizability to various field conditions (see the section of ground removal and bush point denoising in Supplementary Materials).

### Accuracy of size measurements

**Fig. 2:**
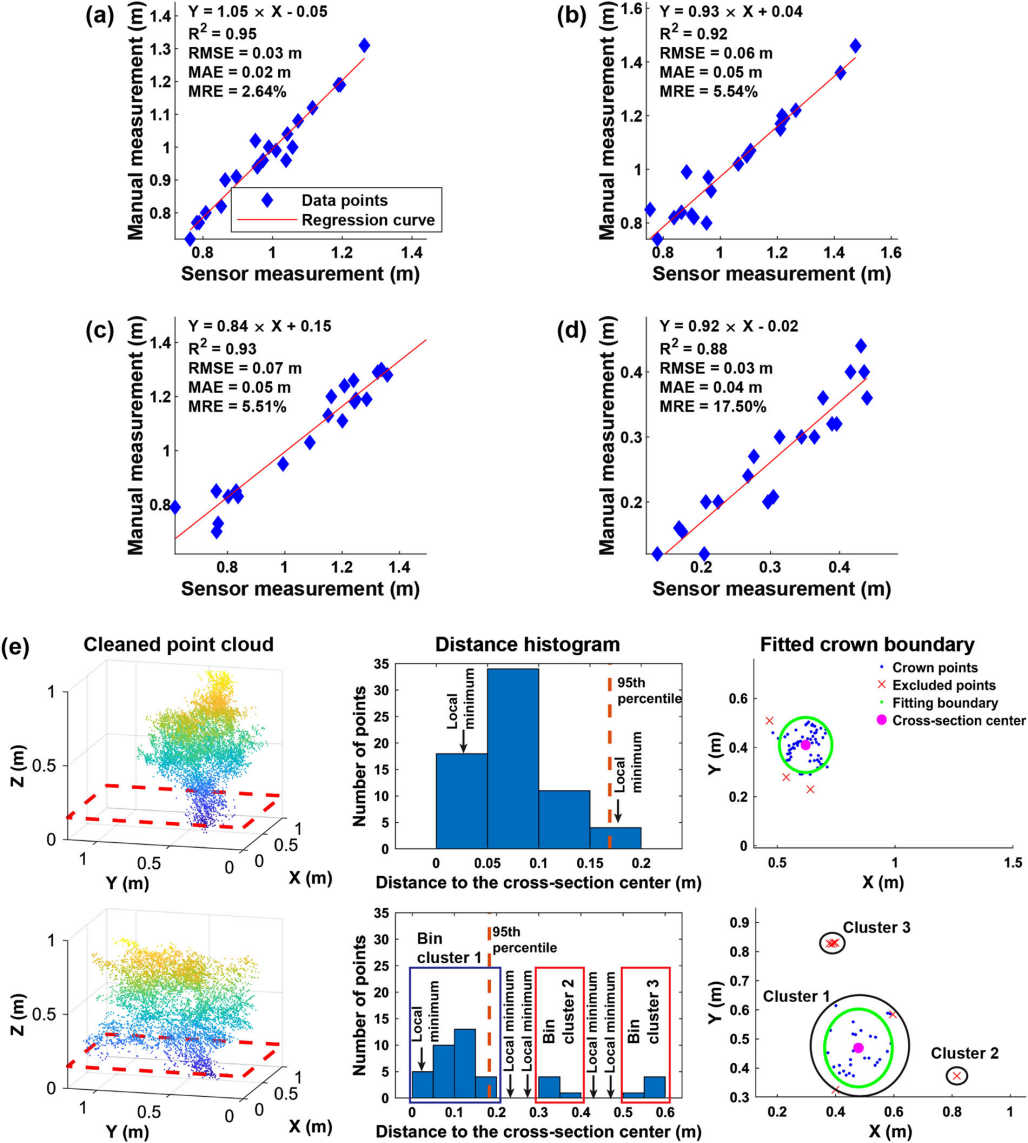
The accuracy of sensor measuremetns and the efficacy of crown size measurements. **a**, **b**, **c**, and **d** are regression results between sensor and manual measurements of bush height, width in row (WIR), width across row (WAR), and crown size; and **e** is the efficacy of the present algorithm for measuring upright (top chart) and inclined (bottom chart) bushes

The scanner achieved the nominal measurement accuracy (2–3 cm) when measuring objects with standard shapes (see the section of validation of measurement accuracy in Supplementary Materials), providing a performance baseline to evaluate accuracies of measuring size-related traits. Generally, correlation was high (*R*^2^ = 0.92–0.95) between sensor and manual measurements of bush height and width (Fig. 2a to Fig. 2c). The RMSE and mean absolute error (MAE) of bush height were comparable with those of objects with standard shapes, whereas the RMSE and MAE of width were two times larger. This occurred primarily because blueberry bushes were non-rigid objects that could be swayed by wind during data collection. Bush movements had relatively small effects on z-direction, resulting in little or no change in height measurements. However, the movements would have substantial effects on the *x*- and *y*-directions, leading to large errors in width measurements. Nonetheless, mean relative errors (MREs) (around 5%) indicated that those errors of width measurements were acceptable.

For bush volume, manual measurements were less than convex hull volumes but greater than concave hull volumes (see Figure S4 (a) and (b) in Supplementary Materials). Compared with concave hull volumes, convex hull volumes were better correlated (*R*^2^ = 0.85) with manual measurements, because both convex hull and manual measurements included space that was not occupied by branches. It was noteworthy that convex hull method included considerably more of unoccupied space between branches than the manual estimation method, resulting in high MAE (0.21 m^3^) and MRE (104%) of volume measurements. In particular, the convex hull method tended to substantially overestimate (around 120%) the volume of bushes with irregular architecture and tall crown (compare (c) and (d) in Figure S4 in Supplementary Materials). This occurred because irregular architecture and tall crown led to large hollow (or empty) areas among (or below) bush canopies that would be included by the convex hull method. However, the manual method used a short height interval (0.05 m in the present study) and significantly reduced the amount of hollow/void areas in volume estimation. Compared with convex hull volumes, concave hull volumes showed a lower correlation with manual measurements, but they were closer to the actual reference values (much smaller MAE (0.05 m^3^) and MRE (19%)). A potential reason was that the amount of space included by the convex hull method was more related to plant size changes than that excluded by the concave hull method. When the plant size increases, the convex hull consistently includes extra space due to the expansion of plant points, but the concave hull method may or may not exclude space depending on the local surface. The concave hull method could match with manual measurements for a flat surface, while it could exclude a large space for a curved surface such as the transition section between canopy and non-canopy parts. The bushes had a large variation of the surface curvature, leading to inconsistent changes of space exclusion by the concave hull method and thus a lower correlation with the plant size changes. For bushes with a relatively regular shape, if point clouds were dense enough, the concave hull volume should be the most accurate measurements; otherwise, it represented the lower limit of bush volume. For instance, if a bush grew in a more regular shape, the concave hull volume was closer to the manual measurement (compare (c) and (d) in Figure S4 in Supplementary Materials).

A high correlation (*R*^2^ = 0.88) was also achieved between sensor and manual measurements of crown size (Fig. 2d). Both RMSE (0.03 m) and MAE (0.04 m) were close to the nominal instrument accuracy, indicating a high measurement accuracy of the present algorithm. Compared with a previous study^27^, the correlation (*R*^2^) increased from 0.56 to 0.88 and the RMSE decreased from 0.06 to 0.03 m, both of which were substantially improved. These improvements were achieved due to the appropriate exclusion of non-crown points (Fig. 2e). For upright bushes, the cross section usually contained one core point cluster with several points that were somewhat away from the cluster (see first row in Fig. 2e). Direct use of the cross-section points would result in a large error of crown size measurements, regardless of using either a fitted diameter or width across-row of the cross section as the crown size. On the contrary, the present algorithm filtered out distant points using the 95th percentile (an empirical value) of distances to the cross-section center, reducing the measurement error. In addition, main bush branches would not naturally distribute as a circle, so an ellipse shape was better for crown fitting and thus crown size measurement. For inclined bushes, the cross section mostly contained several point clusters (see bottom charts in Fig. 2e). The cluster closest to the cross-section center represented the actual crown, whereas the clusters away from the cross-section center were points of branches. Thus, the measurement accuracy was improved by using only the closest cluster.

### Efficacy of crown size and shape descriptors for bush identification

**Fig. 3:**
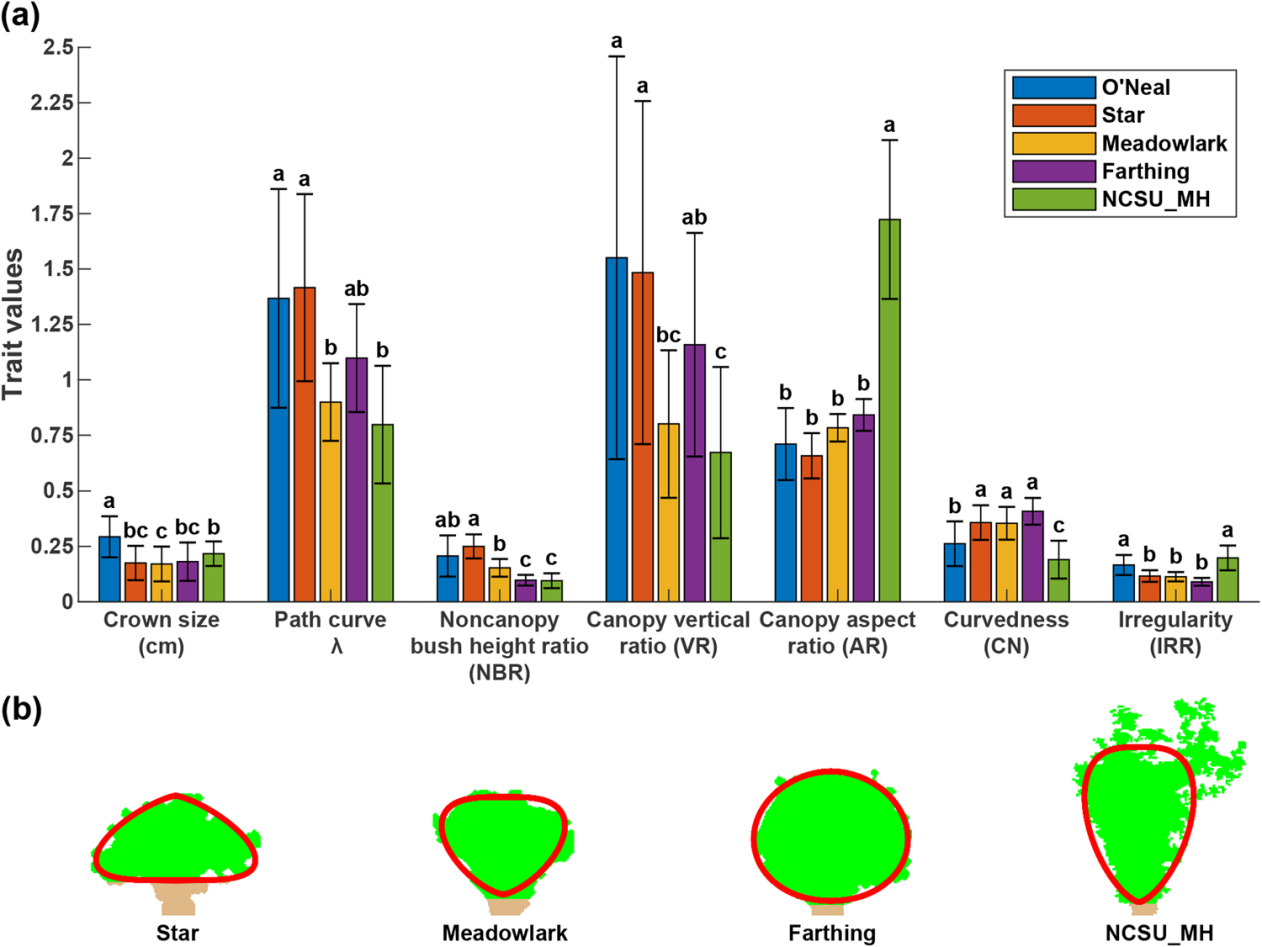
Crown size and shape analysis results of the five blueberry groups. **a** Statistical analysis results of the extracted crown size and shape descriptors and **b** fitted path curves of representative bushes. Groups with different letters are statistically significant with each other, and group mean values of each index are sorted alphabetically. In **b**, green and brown colors are used to render canopy and non-canopy parts of individual blueberry plants, and red curves are the fitted path curves. No representative bush was selected for the O’Neal group due to the large variation of crown size and bush shape in the group

Crown size and shape descriptors showed statistical differences among five genotype groups including four cultivars and one research population bred in North Carolina for mechanical harvesting (NCSU_MH group hereafter) (Fig. 3). The crown size of O’Neal cultivar was statistically more significant than that of the remaining groups, whereas crown sizes of the remaining groups were in a similar range although Meadowlark cultivar had the smallest crown size. This occurred primarily due to two reasons. First, bushes in the five groups were treated with different agronomic practices such as pruning of low-angled branches originating near the ground and large upright canes away from the core cluster. O’Neal bushes were planted in a research farm and not pruned for 2 years before the data collection, resulting in a larger crown size. On the contrary, other group bushes were routinely pruned (based on commercial production guideline) and regulated (only for Star, Meadowlark, and Farthing), leading to a smaller crown size with less variations. Second, the five groups were being evaluated for different breeding targets. The four cultivars were bred primarily for features such as high fruit quality and size, whereas the NCSU_MH group have been selected for mechanical harvesting that requires a small crown. Catch plates on OTR harvesters are pivot mounted on a rail on both sides of the harvester frame and overlap with neighboring plates (see Figure S5 (a) and (b) in Supplementary Materials). When the harvester moves to contact blueberry plants, catch plates are pushed to sides, allowing bush canes to go into the harvester, and then the plates retract to cover empty areas. When catch plates (e.g., fishscales) do not fully retract and return to a crown size area at the base of the plant, it would create an opening area where detached blueberries can potentially fall through to ground (e.g., ground loss). Smaller crown means less ground loss. Thus, the NCSU_MH group should present desired crown size even without crown regulation.

All shape descriptors showed significant differences between at least two genotype groups, suggesting that the shape descriptors could be used for identifying blueberry genotypes with different bush architecture. The NCSU_MH group had the least *λ* values (0.8 ± 0.26), followed by Meadowlark (0.89 ± 0.17), Farthing (1.1 ± 0.24), and Star (1.42 ± 0.42). By definition, these *λ* value ranges indicated in general a vase-shaped canopy for NCSU_MH and Meadowlark groups, a round canopy for Farthing, and a conical canopy for Star (Fig. 3b). Thus, NCSU_MH and Meadowlark bushes would have an optimal shape (vase shape) for mechanical harvesting, which agreed with human subjective evaluation. Meadowlark also can be grafted on sparkleberry (*Vaccinium arboreum*) rootstock with monopodial growth habit, which creates even smaller crown diameter^47^. It should be noted that canopy vertical ratio (VR) showed the same trend and statistical results as *λ*. This was because VR essentially quantified the location of the widest canopy cross section where branches expanded horizontally. An ideal vase shape would have the horizontal expansion at a higher position of the canopy, leading to VR values < 1, whereas a conical shape would have the opposite pattern. A round shape would result in VR values = 1. Although VR showed the same efficacy as *λ* in overall shape quantification, VR values had larger variations than *λ*, which presented a concern of using it for differentiating blueberry genotypes with a small number of replications. The capability of using extracted traits for genotype differentiation needs to be further tested when a smaller number of replications is used.

In contrast to crown size, *λ*, and VR, other shape descriptors (non-canopy-bush height ratio (NBR), canopy aspect ratio (AR), canopy curvedness (CN), and canopy irregularity (IRR)) could not be used for bush shape evaluation based on simple rules (e.g., small crown is preferred), requiring more domain knowledge for proper interpretation and use. Star had the highest NBR value indicating the tallest non-canopy part, which is good for mechanical harvesting due to more positions for configuration of harvester catch plates. However, an excessively tall crown is not desired because it may result in a yield reduction more substantially than the ground loss due to mechanical harvesting. In commercial field setup for mechanical harvesting, low hanging branches are pruned to eliminate their interference with the catch plates and minimize bush crown size to prevent excessive ground loss, but the pruning cannot be aggressive to impact yield. Thus, the NBR index needs to be used as a balance factor for breeding blueberry genotypes suited to mechanical harvesting. AR values of four cultivars were significantly lower than the NCSU_MH group. A low AR value indicates an oblate canopy, which is desired owing to a short dropping height (and thus reduced external impacts) for berries, but if the canopy is excessively oblate, mechanical harvesters may damage branches as well as berries on those branches, decreasing harvest yield and berry quality. It was also noteworthy that the AR index might reflect breeding preferences due to different growing environments. The four cultivars are widely grown in southern Georgia, whereas the NCSU_MH group has been bred in a research station along the coastal area where wind would be generally strong during blueberry vegetative and reproductive growth stages (March–May). Thus, the use of AR index also requires considerations of other factors to evaluate the fitness of bush shape for mechanical harvesting. CN evaluated the curvedness of bush canopy contour and IRR indicated the likelihood of having abnormally extended portions, both of which provided useful information for agronomic management such as pruning. In particular, the IRR values indicated the management practices conducted on bushes. The O’Neal and NCSU_MH groups showed statistically lower CN values and higher IRR values, suggesting a suboptimal bush architecture regarding agronomic management, but they had different reasons: the O’Neal group was due to insufficient management (no pruning for 2 years), whereas the NCSU_MH group was due to different growth periods. Data collection of the NCSU_MH group was conducted in March when bushes had little or no leaf, and consequently bushes were expected to be more irregular. Nonetheless, NBR, AR, CN, and IRR require additional considerations from various aspects for proper interpretation and cannot be used as simple criteria for bush selection and management.

### Visualization tool for identification of optimal bush architecture for mechanical harvesting

**Fig. 4:**
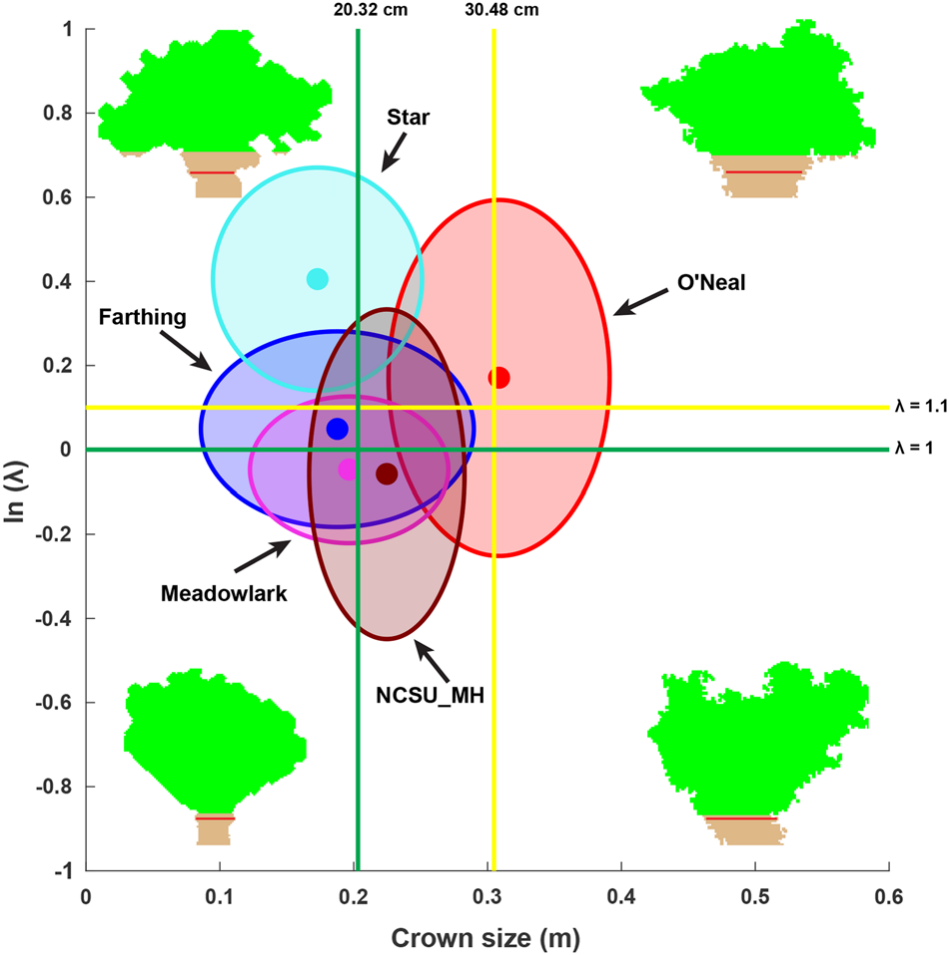
Scatter plot of crown size and the natural logarithm of *λ* for 145 bushes. Green (or yellow) axes indicate the value limits of crown size and path curve *λ* for bushes well suited to (or acceptable for) mechanical harvesting. Solid circles represent the center of individual clusters. For each representative bush, green and brown colors are used to render the canopy and non-canopy parts, and the red line in each bush silhouette indicates the height where the crown size was measured

As the crown size and *λ* could be used for bush evaluation based on simple criteria, a visualization tool (scatter plot) was generated to identify an optimal bush architecture for mechanical harvesting (Fig. 4). Two standard axes were made based on requirements of crown size and bush shape for mechanical harvesting. For an ideal bush architecture, the crown size needs to be less than 20.32 cm^48^ and the bush needs to be vase-shaped (*λ* < 1), whereas for an acceptable bush shape, the crown size can be increased to 30.48 cm^48^ and the bush can be slightly conical (*λ* < 1.1). The two standard axes split the space into four quadrants: (1) the upper left is for bushes with desired crown size but undesired bush shape; (2) the upper right is for bushes with undesired crown size and shape; (3) the lower left is for bushes with desired crown size and bush shape; and (4) the lower right is for bushes with desired shape but undesired crown size. When using the ideal criterion, Meadowlark was the only group having most bushes with ideal crown size and bush shape. When using the acceptable criterion, most Farthing and NCSU_MH bushes met the requirements of crown size and bush shape for mechanical harvesting, whereas the O’Neal group primarily laid in the first quadrant where both crown size and bush shape were not ideal for mechanical harvesting. Star bushes were mostly identified in the second quadrant where the bush shape was not acceptable for mechanical harvesting.

## Discussion

The data processing pipeline has demonstrated the feasibility of using a handheld mobile laser scanner to measure size-related traits and shape descriptors of blueberry bushes in the field. For data collection, the scanner has a dynamic and expansive sensing perspective, which is a major advantage over aerial imaging systems and terrestrial LiDAR systems in which cameras or LiDARs acquire data from an individual angle. The scanner node keeps moving along and across the operator’s movement direction, so a wide range of sensing angles are used to dramatically reduce the possibility of missing points due to object occlusions. However, the scanning throughput of the handheld scanner is relatively low. If the operator keeps oscillating the scanner node and walks at 1.4 m/s (a regular walking pace), the scanning throughput is 0.42 ha/h. Considering the weight of the scanner with necessary accessories (2.5 kg in total weight), operators may become fatigued after collecting data for a period. In practice, it is also difficult for operators to continuously oscillate the sensor node, so oscillating sensor node can be problematic in a long-time data collection session, which reduces the diversity of sensing angles and thus the data quality. To increase the scanning throughput and avoid human fatigue issues, it is necessary to integrate the scanner with motorized vehicles for autonomous data collection. In fact, the scanner was originally developed for both handheld and vehicle-based applications^31^, so it can be mounted on a motorized platform (e.g., a gator utility vehicle) with modifications to improve the data collection throughput.

The present data processing pipeline can accurately extract size-related traits, especially the crown size. Compared with a previous study^27^, the measurement accuracy of crown size has been significantly increased due to not only the improved measurement algorithm but also a different way of collecting point cloud data. As for the aforementioned advantage, the scanner can have various sensing angles, and some angles (e.g., parallel with bush crown) can be particularly useful for acquiring points of the bush crown that is usually occluded by bush canopies from the nadir and top-to-bottom oblique views. If raw point clouds miss many points of the bush crown (or other bush parts), it is not possible to improve the measurement accuracy of algorithms. Although the processing pipeline is independent of data collection systems, the processing performance highly depends on the quality of acquired data that are affected by data collection systems to a certain extent. Due to practical reasons (e.g., easy to measure ground truth data), small to mid-size bushes were used to evaluate the accuracy of the presented approach, which avoided a potential issue of branch entanglement between neighboring plants. The entanglement usually introduces difficulties in accurate segregation of individual plants (especially the upper canopy), which could dramatically affect the measurement of width in-row (WIR) and bush volume.

The extracted crown size and shape descriptors (primarily *λ*) provide objective evaluation and measurements for identifying bushes suitable for mechanical harvesting. In particular, the visualization tool (scatter plot of crown size and *λ*) is particularly useful for rapid determination of optimal bush architecture. NBR and AR indices can be used as balance factors to select bushes suited to a particular harvesting machine or growing environment and maintain other desired features such as yield. CN and IRR can be incorporated into agronomic management decision process such as pruning. However, the use of the four parameters (NBR, AR, CN, and IRR) highly depends on breeding and management purposes. Thus, thresholds or value ranges of the parameters for optimal bush architecture need to be determined with specific domain purposes and may vary dramatically among applications. In addition, all the extracted traits could be used by harvester manufacturers to improve the design of fruit catching system. The five genotype groups were selected because they had distinctive bush architecture. With a large number of replications (at least 20 reps per group), it would be relatively easy to differentiate the groups from each other using the crown size and shape descriptors. We acknowledge that it is necessary to conduct successive studies involving a wide variety of genotypes with fewer replications, so the statistical power of extracted traits can be further tested for genotype differentiation.

## Conclusions

The data processing pipeline presented in this study accurately measured size-related traits and bush shape from point cloud data collected by the handheld mobile laser scanner in the field. Shape descriptors were used to identify bushes with desired features for machine harvesting, and bushes with non-ideal shapes that required pruning actions. Thus, the present processing pipeline with the data collection instrument is particularly useful for blueberry breeding programs and farm management. Future studies will focus on the development of autonomous data collection system and experiments of using shape descriptors for genotype differentiation in a large-scale field.

## Materials and methods

### Blueberry field and data collection

The study was conducted in two blueberry fields. The first field (33°53′10.7″N and 83°25′15.1″W) was located at the Horticultural Farm of the University of Georgia in Watkinsville, Georgia, USA and consisted of 7-year-old southern highbush blueberry (*Vaccinium darrowii*) bushes. The bushes had been un-pruned for 2 years at the time of this study. Point cloud data were collected in a sub area (23 m × 15 m) containing 47 bushes (O’Neal cultivar) on 4 October 2016 with a clear sky view and an average wind speed of 2.7 m/s. A closed-loop walking path was predetermined, with at least one pass for each side of individual bushes. This walking strategy ensured that bushes would be scanned from multiple angles to improve point cloud coverage. While moving along the predefined path, a person carried the ZEB1 scanner (GeoSLAM, Ruddington, Nottinghamshire, United Kingdom) and swung the scanner node across the movement direction. The walking speed was about 1.4 m/s, and it took approximately 5 min to complete the scanning. A total of 20 bushes were selected for further analyses, because they were relatively small plants, which in practice could be measured manually.

The second field (34°21′42″N and 77°50′11.9″W, 68 m × 12.5 m) named Ideal Tract Farm was in the Horticulture Research Station in North Carolina. The study characterized 222 8-year-old bushes that have been bred for mechanical harvesting. The 222 bushes were pruned prior to data collection. Data collection was conducted on 15 March 2018 with an overcast sky and an average wind speed of 3.5 m/s. A closed-loop walking path was used with one pass on each side of individual bushes, ensuring that bushes would be scanned from multiple angles to improve point cloud coverage. While moving along the predefined path, a person carried the ZEB1 scanner and swung the scanner node across the movement direction. The walking speed was about 1.0–1.2 m/s due to the muddy ground condition, and it took approximately 10 min to complete the scanning. All 222 bushes were used for further data analyses, but no manual measurements were conducted.

### Data processing pipeline of extracting size and shape traits

#### Point cloud preprocessing

**Fig. 5:**
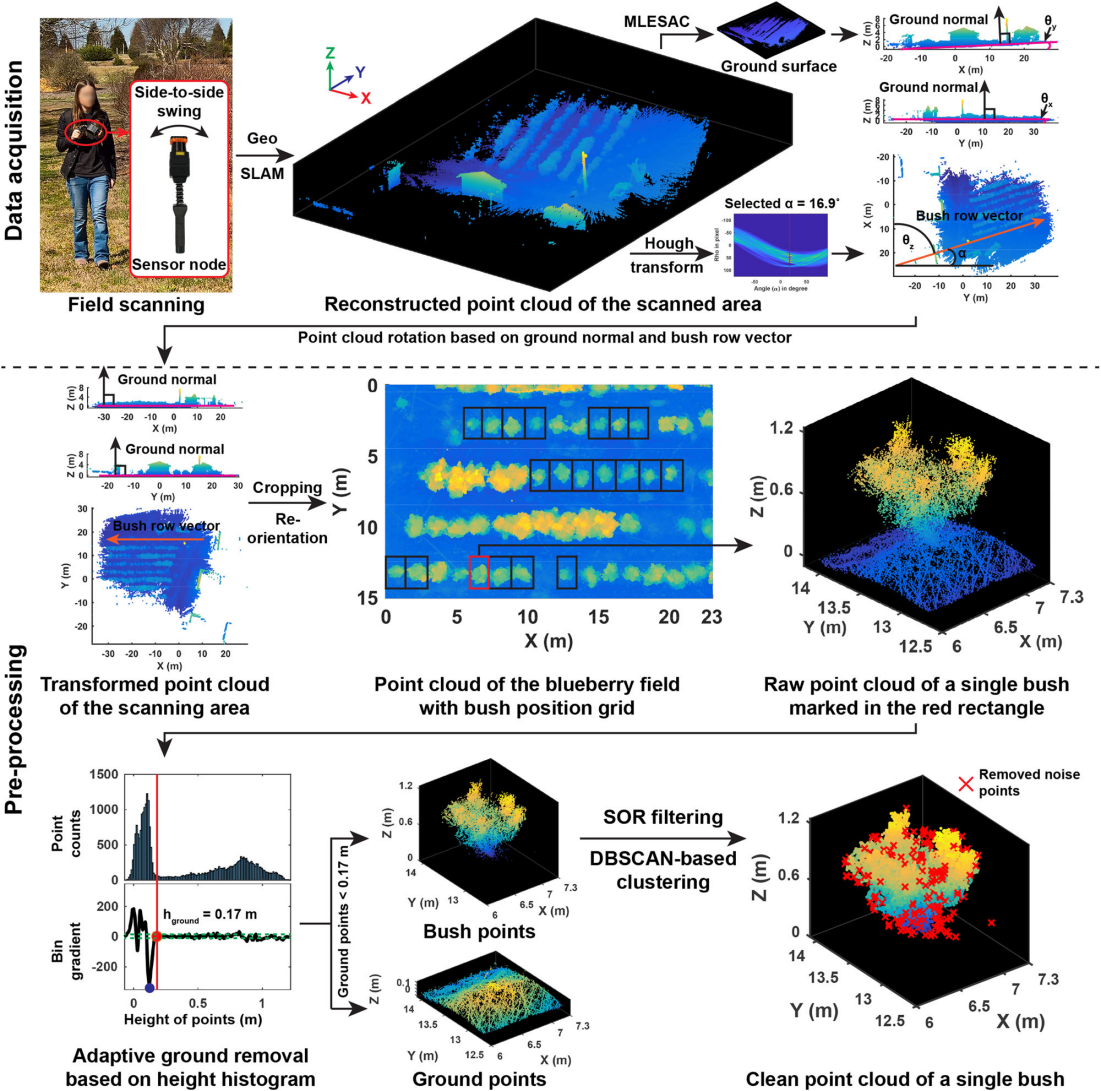
Flowchart of data acquisition and preprocessing to obtain clean point clouds of individual blueberry bushes. In the diagram, for the adaptive ground removal based on height histogram, the red and blue dots indicated the determined threshold and the height value with the least bin gradient, respectively. The green dashed lines depicted the value range in which bin gradient values were close to zero

**Fig. 6:**
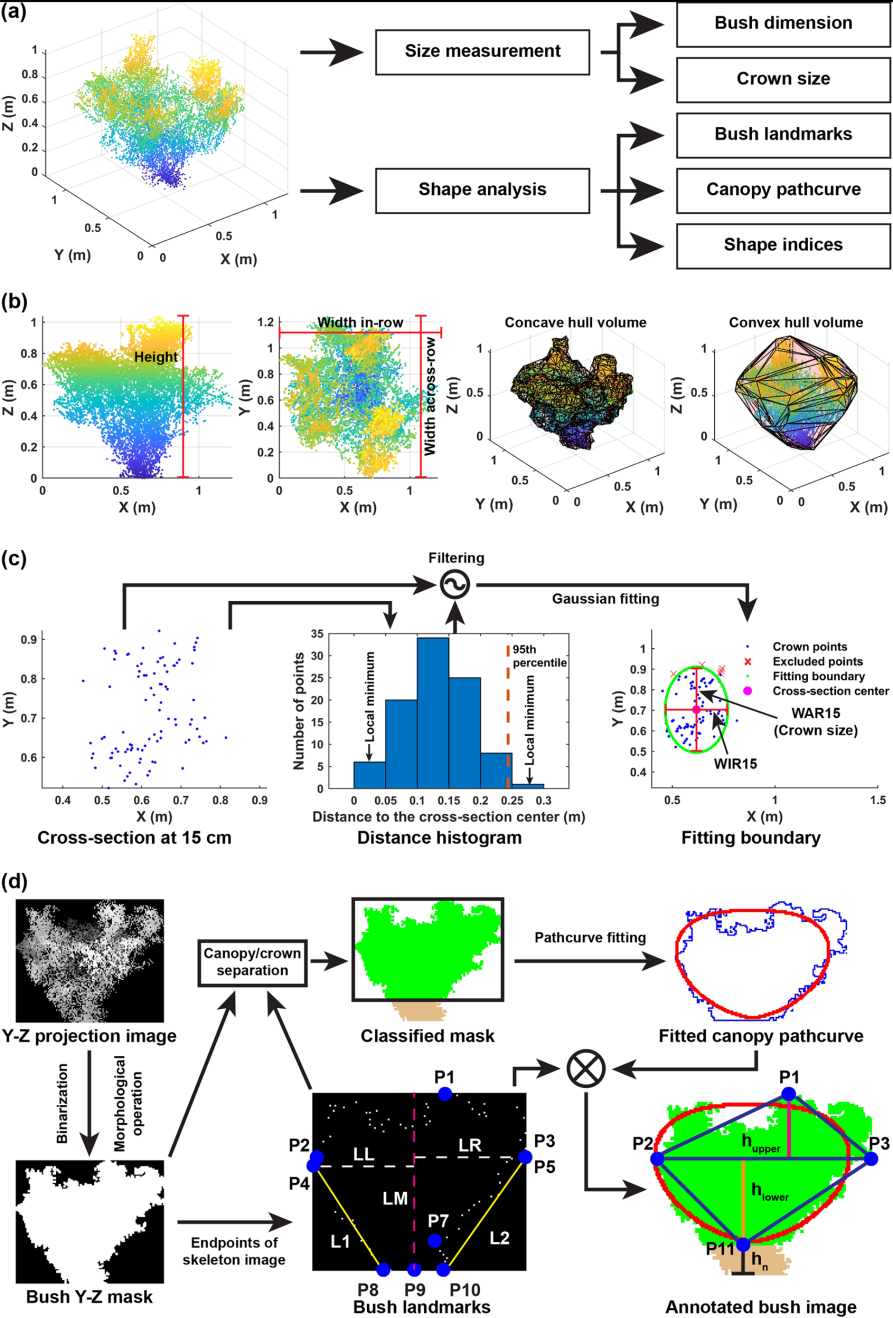
Flowchart of data processing to extract size and shape traits for blueberry bushes. **a** Overall diagram of trait extraction; **b** measurement of size-related traits; **c** measurement of bush crown size; and **d** calculation of bush shape indices. In **c**, WAR15 and WIR15 were the width across-row and width in-row of the cross section at 0.15 m above the ground, and WAR15 was used as crown size in the present study. In **d**, green and brown colors indicated the canopy and crown part of bush. Blue and red curves were the contours and the best fitted path curves of the bush canopy. P1 to P11 denoted 11 landmark points including the bush top-center point (P1), left (P2) and right (P3) end points of the broadest cross section, bush leftmost (P4) and rightmost (P5) end points, left (P6) and right (P7) canopy-crown separation points, left (P8), center (P9), and right (P10) end points of the bush bottom, and the center point of the canopy bottom (P11). It should be noted that in this case, P6 did not exist and P3 and P5 overlapped. LM was the center line of the bush, and LL and LR were the left and right border lines between bush upper and lower portions. L1 and L2 were the left and right outer boundary lines of the bush bottom portion. *h*_n_, *h*_upper_, and *h*_lower_ denoted the height of the bush crown and canopy upper and lower triangles

A data processing pipeline was developed to extract size-related traits and bush shape, including data acquisition, preprocessing, and trait extraction (Figs. 5 and 6). Raw data were manually transferred from the scanner to a workstation computer and uploaded to the manufacturer’s web service (GeoSLAM Cloud, Bingham Nottingham, Notts, UK) for 3D reconstruction. The reconstructed point cloud data contained 4.7 million points (152 MB in LAS format) and 17 million points (547 MB in LAS format) for the two experimental fields.

In the preprocessing stage, clean point clouds of individual blueberry bushes were obtained (Preprocessing in Fig. 5). The first step was to rotate the point cloud of the entire scanning area to a coordinate system in which the ground plane was paralleled to the *x*-*y* plane and the bush row direction was aligned with *x*-positive direction. Ground normal was calculated through plane fitting using maximum likelihood estimation sample consensus^49^, and rotation transform matrices (*T*_*y*_ and *T*_*x*_) were accordingly derived for the *x*-*z* and *y*-*z* planes. Rotation matrix for the *x*-*y* plane (*T*_*z*_) was derived based on the row direction identified using Hough transform. The point cloud of the scanning area was rasterized to a depth image, with each image pixel representing the maximum depth (*z* value) in a grid of 0.5 × 0.5 m^2^ in the point cloud. The depth image was thresholded (the threshold was 0.3 because bushes were at least 0.3 m above the surrounding ground) to segment bush pixels. The Hough transform was performed on all bush pixels to detect lines (bush rows) in a feature space ([*α, ρ*]) where *α* was the complementary angle to the line orientation and *ρ* was the distance from the line to the origin. If a line represented a bush row, the line should go through bush pixels as many as possible, and thus the high occurrence of [*α, ρ*] values (the *α* line with the least intersection points with curves in the Hough space graph) denoted bush rows. The best *α* value was accordingly selected to calculate the bush row direction. Subsequently, a rotated point cloud of the scanning area was calculated using Eq. 1.

1 $${\mathrm{PtCloud}}_{{\mathrm{rotated}}} = {\mathrm{PtCloud}}_{{\mathrm{orign}}}T_xT_yT_z$$

$$\begin{array}{l}T_x = \left[ {\begin{array}{*{20}{c}} 1 & 0 & 0 \\ 0 & {\cos \theta _x} & {\sin \theta _x} \\ 0 & { - \sin \theta _x} & {\cos \theta _x} \end{array}} \right]\\ T_y = \left[ {\begin{array}{*{20}{c}} {\cos \theta _y} & 0 & {\sin \theta _y} \\ 0 & 1 & 0 \\ { - \sin \theta _y} & 0 & {\cos \theta _y} \end{array}} \right]\\ T_z = \left[ {\begin{array}{*{20}{c}} {\cos \theta _z} & {\sin \theta _z} & 0 \\ { - \sin \theta _z} & {\cos \theta _z} & 0 \\ 0 & 0 & 1 \end{array}} \right]\end{array}$$

Where PtCloud denoted a point cloud matrix, *T* represented rotation matrices, and *θ*_*x*_, *θ*_*y*_, and *θ*_*z*_ were rotation angles around the *x*-, *y*-, and *z*-axes, respectively.

The rotated point cloud was re-oriented to ensure that the bush row vector was toward the *x*-positive direction, and then the experimental area was cropped based on its dimension (23 and 15 m along *x*-positive and *y*-positive directions). Bounding boxes were manually drawn for the selected 20 bushes, and raw point clouds of individual bushes (PtCloud_RawBush_) were segregated accordingly.

The second step was to remove ground and noise points in raw bush point clouds, obtaining clean bush point clouds for trait extraction. The ground surface was not flat due to agronomic practices (e.g., additional woody layer), and as a result plane-fitting-based methods such as random sample consensus could not remove ground points correctly. An adaptive thresholding approach was proposed for ground point removal. In each raw bush point cloud, a height histogram was generated using a bin width of 0.01 m, and bin gradients were calculated accordingly. The threshold of ground points was determined by three criteria: its bin gradient was close to zero; it was greater than the lower limit of the bin with the least gradient value; and it should be as small as possible. The threshold was calculated using Eq. 2.

2 $${h_{{\mathrm{ground}}} = \min \left( {\left\{ {h\,{\mathrm{|}}\,0 < \left| {G\left( h \right)} \right| < t} \right\} \cap \left\{ {h{\mathrm{|}}h > {\mathrm{argmin}}_h(G(h))} \right\}} \right)}$$

where *h*_ground_ was the determined height threshold for ground points, *G*(*·*) was the gradient of a bin, *h* was the lower limit of bins in a height histogram, and *t* (set to 5 in the present study) was a noise factor for selecting bins with the gradient close to zero.

If point heights were lower than the threshold, the points in raw bush point clouds (PtCloud_RawBush_) were classified as ground (PtCloud_ground_) or otherwise bush (PtCloud_bush_). After removing ground points, noise points in bush point clouds (PtCloud_bush_) were detected and excluded using statistical outlier removal (SOR) filter. For each point, Euclidean distances to its *k* nearest-neighboring points were calculated. A point was categorized as noise, if the mean distance between that point to its *k* neighboring points was larger than *n* times of the standard deviation (Eqs. 3 and 4).

3 $$D\left( {p,k} \right) = \frac{{\mathop {\sum }\nolimits_{i = 1}^k \sqrt {(p_x - p_x^i)^2 + (p_y - p_y^i)^2 + (p_z - p_z^i)^2} }}{k}$$

4 $$N\left( p \right) = \left\{ {\begin{array}{*{20}{c}} {1\left( {{\mathrm{noise}}} \right),} & {\mu _{D\left( {p,k} \right)} > n \times \delta _{D(p,k)}} \\ {0,} & {{\mathrm{otherwise}}} \end{array}} \right.$$

where *D*(*p*, *k*) denoted a range of Euclidean distances between a point *p* in bush point clouds to each of its *k* nearest neighbors. _*pi*_ indicated the *i*th neighboring point of *p*. *p*_*x*_, *p*_*y*_, and *p*_*z*_ ($p_x^.$, $p_y^.$, and $p_z^.$) were the *x*, *y*, and *z* coordinates of the point *p* (or its neighboring point ^*pi*^) in point clouds. *N* denoted noise flag, and *μ*_*D*_ and *δ*_*D*_ were the mean and standard deviation of *D*(*p*, *k*), and *n* was the scalar of standard deviation.

Based on some preliminary tests, *k* and *n* were set as 10 and 1 in this study, respectively. The SOR filter could eliminate scattered noise points but not point clusters of relatively large objects such as weeds. Density-based spatial clustering of applications with noise (DBSCAN) algorithm was used to further filter out point clusters of non-bush objects. After SOR filtering, the points were clustered using the DBSCAN algorithm, and the largest point cluster was selected as the clean bush point cloud (PtCloud_CleanBush_) (Eq. 5).

5 $${\mathrm{PtCloud}}_{{\mathrm{CleanBush}}} = {\mathrm{argmax}}_C\left( {\left| {C_i} \right|} \right),i = 1,2, \ldots ,n$$

where PtCloud_CleanBush_ denoted the clean point cloud of a blueberry bush, *C* denoted a point cluster that was identified using the DBSCAN algorithm, *i* was the index of identified point clusters, ranging from 1 to *n*, and |·| operator calculated the number of points in a point cluster.

#### Size-related trait measurement

Size-related traits were measured from the clean bush point clouds of individual bushes (Fig. 6a). The measurement was to calculate bush dimensions and crown size, and the shape analysis was to find the best boundary curve of canopy and derive shape indices.

##### Bush dimension

Bush dimension parameters included bush height, WIR, and width across-row (WAR), which were the maximum length of a bush along the *z*-, *x*-, and *y*-directions, respectively (Fig. 6b). Bush volume was estimated using concave and convex hulls.

##### Crown size

Crown size is an essential dimensional parameter, affecting the configuration of catch plate (also known as fish scale) and ultimately the performance (e.g., ground loss) of machine harvesters. In the horticultural community, the term “crown” refers to a cross section at a certain height^50^. In this study, crown size was defined as the bush diameter across-row at 15 cm from the bottom of main stems where the catch plates of OTR harvesters contact with plants. Cross-section points at such height were separated using height information, and subsequently distances from individual cross-section points to the cross-section median center were calculated (Fig. 6c). A distance histogram was generated with a bin width of 0.05 m, and local minimal bin values (the bin value is less than that of two neighboring bins) were identified to group bins into different bin clusters. The first bin cluster contained points representing the bush crown, and the 95th percentile distance of the first bin cluster (between the first and second local minimal bin values) was used as the threshold to exclude cross-section points that were far away from the cross-section center. The retained points (*P*_retained_) were fitted to a 2D Gaussian distribution. The distribution mean and variances were used as the center and initial values of the semi-axes for an ellipse curve. Constrained optimization was used to find the minimum values of the two axes, so that the ellipse curve could reach a predefined point coverage (Eq. 6). The ellipse’s vertical diameter was the crown size of a bush.

6 $$\begin{array}{*{20}{c}} {{\mathrm{min}}} & {f\left( {d_x,d_y} \right) = d_x + d_y} \\ {{\mathrm{subject}}\,{\mathrm{to}}} & {\frac{{|P_{{\mathrm{covered}}}|}}{{|P_{{\mathrm{retained}}}|}} \ge T_{{\mathrm{coverage}}},P_{{\mathrm{covered}}} = \left\{ {p{\mathrm{|}}\frac{{p_x^2}}{{d_x^2}} + \frac{{p_y^2}}{{d_y^2}} \le 1,p \in P_{{\mathrm{retained}}}} \right\}} \end{array}$$

where *d*_*x*_ and *d*_*y*_ were the horizontal and vertical diameters of the ellipse curve. *T*_coverage_ was the predefined coverage (set as 0.9 in the present study). *P*_covered_ is a set of points covered by the fitted elliptical curve and *p* indicated a point in the two sets (*P*_covered_ and *P*_retained_). |·| operator calculated the number of points in a point set.

#### Bush shape analysis

Due to the importance of machine harvester configuration and performance, the across-row bush shape was analyzed in the present study (Fig. 6d). For each bush, clean bush point cloud was projected onto the *y*-*z* plane, and rasterized to a grayscale image using a grid size of 0.01 × 0.01 m^2^. As the shape analysis was conducted on images, the coordinate system used the top-left corner as the origin (0, 0) and *x*- and *y*-coordinates increased along the right and downward directions. In the grayscale image, pixel intensity represented the distance from a pixel to the starting point of a bush along the *x*-direction. The grayscale image was thresholded to a raw bush mask, and morphological operations were used to remove noise pixels and fill holes, generating the final bush mask for successive processing.

##### Landmark point detection

A total of 11 landmark points were defined in the present study, including the center point of the bush topmost row (P1), left (P2), and right (P3) end points of the broadest horizontal cross section, bush leftmost (P4) and rightmost (P5) end points, left (P6) and right (P7) canopy-crown separation points, left (P8), center (P9), and right (P10) end points of the bush bottom, and the center point of the canopy bottom (P11). P1 to P5 and P8 to P10 were detected based on their definitions, and five lines (LM, LL, LR, L1, and L2) were identified accordingly. LM was the center line of bush, which is a vertical line passing through P11. LL was the left border lines, which is a horizontal line that passes through the point close to the bush bottom in P2 and P4 and intersects with LM. L1 was the left outer boundary passing through P8 and the point close to bush bottom in P2 and P4. Similarly, LR and L2 were identified on the right side of the bush. LM, LL, and LR split the skeleton end points into four quadrants. Distances from individual end points in the left-lower quadrant to L1 were calculated. P6 was an end point in the left-lower quadrant that satisfied four criteria: (1) it had the most considerable distance to L1; (2) its distance to L1 was larger than a threshold (the median of all left-lower end points’ distances plus the median absolute deviation (MAD)); (3) it was close to the bush bottom as much as possible; and (4) it located above L1 (Eqs. 7 and 8).

7 $$P6 = px_{k},k = {{\rm argmax}}\left( {px_{i,n}} \right)\,{{\rm and}}\,px_i \in P_{{{P6{\rm candidate}}}}$$

8 $$\begin{array}{l}P_{{{P6{\rm candidate}}}} = \left\{ {px_j{\mathrm{|}}D_{ll}\left( {px_j} \right) = \max \left( {D_{ll}} \right) \cap D_{ll}\left( {px_j} \right)} \right.\\ \left. { > {\mathrm{Med}}\left( {D_{ll}} \right) + {\mathrm{MAD}}\left( {D_{ll}} \right) \cap px_{j,n} > f_{L1}(px_{j,m})} \right\}\end{array}$$

Where *px* indicated an end-point pixel in the lower-left quadrant, and *k*, *i*, and *j* were the indices of P6 pixel, P6 candidate pixels (*P*_P6candidate_), and end-point pixels in the lower-left quadrant, respectively. *px*_*j, m*_ and *px*_*j, n*_ were the horizontal and vertical coordinates of a pixel *px*_*j*_ in images. *D*_*ll*_ denoted the set of distances from individual left-lower end points to L1. *Med* and *MAD* were operators to calculate the median and MAD values of a set. *f*_L1_ was the function of L1.

P7 could be identified using the same criteria in the lower-right quadrant. The vertical coordinate of the one close to the bush bottom in P6 and P7 was used to separate bush crown and canopy. P11 was the center point of the separation cross section. It should be noted that P6 and P7 are not guaranteed to be present, because bush main stems may spread at a position very close to the ground or form branches at higher positions, resulting in an unclear separation between canopy and crown. If both P6 and P7 were missing, the canopy-crown separation line would merge with the bush bottom line, and consequently P11 became the same with P9.

##### Canopy contour fitting

Canopy shape is another important factor affecting the performance of machine harvesting. Vase-shaped canopy is likely to reduce the total harvesting loss and bruising damage, leading to an improved harvest yield and quality. For a blueberry bush, canopy pixels were segmented in the bush mask image using the landmarks, and canopy contour was extracted from the segmented part. Path curve was used to quantify the canopy contour shape. A path curve is defined by a single parameter *λ*: the path curve is a circle when *λ* = 1, and becomes conical (or vase-shaped) when *λ* is larger than 1 (or <1). As the curve position was considered, the function for drawing a 2D path curve was defined using Eq. 9.

9 $$f_{w_{{\mathrm{ptc}}},h_{{\mathrm{ptc}}},\lambda }^{{\mathrm{ptc}}}:t \in {\Bbb R} \to S_{{\mathrm{ptc}}} \in {\Bbb R} \times {\Bbb R}$$

$$\begin{array}{l}f_{w_{{\mathrm{ptc}}},h_{{\mathrm{ptc}}},\lambda }^{{\mathrm{ptc}}}\left( t \right) = a_{{\mathrm{ptc}}}.\left( { \mp w_{{\mathrm{ptc}}}^\prime + {\mathrm{offset}}_m,\frac{{e^th_{{\mathrm{ptc}}}^2}}{2} + {\mathrm{offset}}_n} \right)\\ a_{{\mathrm{ptc}}} = \frac{1}{{e^{ - \lambda t} + \frac{{h_{{\mathrm{ptc}}}}}{2}e^t}}\\ w_{{\mathrm{ptc}}}^\prime = \frac{{w_{{\mathrm{ptc}}} \times \left( {e^{ - \lambda t_{{\mathrm{max}}}} + \frac{{h_{{\mathrm{ptc}}}}}{2} \times e^{t_{{\mathrm{max}}}}} \right)}}{2}\\ t_{{\mathrm{max}}} = \frac{{\ln \left( {\frac{{2\lambda }}{{h_{{\mathrm{ptc}}}}}} \right)}}{{\lambda + 1}}\\ t = [ - 20,20]\end{array}$$

where *f*^ptc^ denoted the function for drawing a 2D path curve, and *w*_ptc_, *h*_ptc_, and *λ* were the width, height, and shape factor of a path curve. $w_{{\mathrm{ptc}}}^\prime$ was the base value of horizontal coordinates of a path curve given the width of *w*_ptc_. *a*_ptc_ reached its maximum value when *t* was *t*_max_. Based on previous study^40^, the domain of definition from −20 to 20 provided adequate range for covering typical object contours.

Detailed mathematical explanations of a typical function of path curve can be found in refs. ^40,46^. To evaluate the fitness of a path curve to a bush canopy contour, an energy function was defined as Eq. 10.

10 $$e_{{\mathrm{ptc}}} = \frac{{\sqrt {\mathop {\sum }\nolimits_{i = 1}^n {\mathrm{dist}}(px_i^{{\mathrm{ptc}}})} }}{n}$$

where *e*_ptc_ was the energy function of a path curve, $px_i^{{\mathrm{ptc}}}$ denoted the *i*th pixel in a path curve, and *n* was the total number of pixels in a path curve. ${\mathrm{dist}}(px_i^{{\mathrm{ptc}}})$ was a function to calculate the distance from $px_i^{{\mathrm{ptc}}}$ to the nearest pixel of the canopy contour.

Gradient descent approach was used to find the optimal path curve parameters (*w*_ptc_, *h*_ptc_, and *λ*) that minimized the energy function. In the present study, the gradient descent optimization would stop, if the path curve energy reached to a minimum value with no change in the following 5 iterations or the total iteration reached 500. The path curve with the minimum energy value was selected as the best-fitting curve using certain initial values. To avoid fitting to local optima, *w*_ptc_ and *h*_ptc_ were initiated with various values. *w*_ptc_ ranged from $\frac{{w_{{\mathrm{canopy}}}}}{2}$ to *w*_canopy_, and *h*_ptc_ ranged from $\frac{{h_{{\mathrm{canopy}}}}}{2}$ to *h*_canopy_, with an increment of 5 pixels for both. *w*_canopy_ and *h*_canopy_ were the width and height of the bounding box of canopy. The path curve with the lowest energy value among the best path curves using various initial values was selected as the final fitting path curve of canopy for a bush.

*Shape index calculation:* The detected landmark points and fitted path curve were used to derive five shape indices: (1) NBR, (2) VR, (3) AR, (4) CN, and (5) IRR (Fig. 6d). The five indices quantified bush shape aspects for machine harvesting and agronomic management. NBR was to evaluate the potential of catch plate configuration, with high values for a wide range of placing catch plates of machine harvesters. VR and AR represented the canopy overall shape. Low VR and AR values (<1) would represent a vase-shaped canopy with short fruit dropping height, which is preferred for machine harvesting; whereas, high VR and AR values (>1) would represent a conical canopy with greater fruit dropping height, which is not ideal for machine harvesting. CN and IRR were more related to agronomic management. In particular, high IRR values indicated an irregular canopy shape that requires agronomic actions such as pruning. Mathematical definitions of the five indices were provided in Eq. 11.

11 $${\mathrm{NBR}} = \frac{{h_{\mathrm{n}}}}{{h_{{\mathrm{bush}}}}} = \frac{{h_{\mathrm{n}}}}{{h_{\mathrm{n}} + h_{{\mathrm{lower}}} + h_{{\mathrm{upper}}}}}$$

$$\begin{array}{l}{\mathrm{VR}} = \frac{{h_{{\mathrm{upper}}}}}{{h_{{\mathrm{lower}}}}}\\ {\mathrm{AR}} = \frac{{h_{{\mathrm{ptc}}}^{{\mathrm{fitted}}}}}{{w_{{\mathrm{ptc}}}^{{\mathrm{fitted}}}}}\\ {\mathrm{CN}} = \frac{{\left| {S_{{\mathrm{ptc}}}^{{\mathrm{fitted}}}} \right| - \left| {S_{{\mathrm{triangles}}}} \right|}}{{\left| {S_{{\mathrm{ptc}}}^{{\mathrm{fitted}}}} \right|}} = \frac{{\left| {S_{{\mathrm{ptc}}}^{{\mathrm{fitted}}}} \right| - \left| {S_{{\mathrm{upper}}}} \right| - \left| {S_{{\mathrm{lower}}}} \right|}}{{\left| {S_{{\mathrm{ptc}}}^{{\mathrm{fitted}}}} \right|}}\\ {\mathrm{IRR}} = \frac{{\left| {{\mathrm{C}}S_{{\mathrm{ptc}}}^{{\mathrm{fitted}}}} \right|}}{{\left| {S_{{\mathrm{canopy}}}} \right|}} = \frac{{\left| {S_{{\mathrm{canopy}}}} \right| - \left| {S_{{\mathrm{ptc}}}^{{\mathrm{fitted}}}} \right|}}{{\left| {S_{{\mathrm{canopy}}}} \right|}}\end{array}$$

where *h*_n_, *h*_upper_, and *h*_lower_ were heights of non-canopy part, and canopy upper (with vertices of P1, P2, and P3) and lower (with vertices of P11, P2, and P3) triangles. $h_{{\mathrm{ptc}}}^{{\mathrm{fitted}}}$ and $w_{{\mathrm{ptc}}}^{{\mathrm{fitted}}}$ were the height and width of the fitted canopy path curve. *S*_upper_, *S*_lower_, $S_{{\mathrm{ptc}}}^{{\mathrm{fitted}}}$, and *S*_canopy_ denoted sets of pixels within the canopy upper and lower triangles, fitted path curve, and the canopy contour. |·| operator calculated the number of pixels in a given set.

#### Performance evaluation

It is important and necessary to evaluate the accuracy of measured size-related traits by the proposed method. Five size-related traits were manually measured for reference, including bush height, WIR, WAR, volume, and crown size. Bush height, WIR, WAR, and crown size were measured using a measuring tape based on their definitions, whereas bush volume was estimated using a cylindrical model (see Figure S6 in Supplementary Materials). A bush was visually and vertically segregated into layers with an interval of 5 cm, with each layer being assumed as a cylinder. Circumference of each layer was manually measured, and thus diameter could be estimated to calculate the layer volume. The summation of all layer volumes was used as a reference value of bush volume. Simple linear regression analyses were performed between sensor and manual measurements for size traits. *R*^2^ and RMSE were used as indicators to evaluate the accuracy of sensor measurements. In addition, MAEs and MREs were calculated as additional parameters for accuracy evaluation. All analyses were conducted in MATLAB (Statistics Toolbox 2017b, The MathWorks Inc. Natick, Massachusetts, USA).

Statistical analyses were conducted on the extracted crown size and shape descriptors to evaluate their usefulness of identifying optimal bush architecture suitable for mechanical harvesting. Although there were 16 genotypes in the North Carolina field, they have been selected for mechanical harvesting and are extremely similar in terms of bush architecture. Thus, it was reasonable to treat all genotypes in the North Carolina field as one genotype group (hereafter, NCSU_MH group). An additional point cloud dataset was used to increase the diversity of bush architecture, containing three highbush blueberry cultivars (Star, Meadowlark, and Farthing) with distinctive bush shapes^27^. In summary, crown size and shape descriptors were extracted using 367 bushes from five genotype groups (20 bushes in the O’Neal group, 222 in NCSU_MH, 42 in Star, 43 in Meadowlark, and 40 in Farthing). As sample sizes were dramatically different among groups, Kruskal-Wallis tests (nonparametric equivalent to analysis of variance test) were performed on extracted crown size and shape descriptors to identify statistical differences among the five groups. Kruskal-Wallis tests were conducted in R 3.4.2 (package asbio) using a significance level of 0.05.

## Code availability

Computer program codes and raw data used in this study can be accessed through https://figshare.com/s/2abb4eeadfda4103545b.

## Supplementary information

Supplementary Materials

## Data Availability

The authors declare that all data supporting the findings of this study are available within the paper and its supplementary information files.
