# Supplementary material for: 3D point cloud data to quantitatively characterize size and shape of shrub crops
Source: Hortic Res. 2019 Apr 6;6:43. doi: 10.1038/s41438-019-0123-9 (PMC6441659; doi:10.1038/s41438-019-0123-9)
Supplement: Supplementary file 1 — Supplementary Materials [file 41438_2019_123_MOESM1_ESM.docx]

**3D point cloud data to quantitatively characterize size and shape of shrub crops**

**Supplementary Materials**

Yu Jiang^1^, Changying Li^1,*^, Fumiomi Takeda^2^, Elizabeth A. Kramer^3^, Hamid Ashrafi^4^, and Jamel Hunter^5^

1. School of Electrical and Computer Engineering, College of Engineering, The University of Georgia, Athens, Georgia 30602, United States of America

2. Appalachian Fruit Research Station, United States Department of Agriculture-Agricultural Research Service, Kearneysville, West Virginia 25430, United States of America

3. Department of Agricultural and Applied Economics, College of Agricultural and Environmental Sciences, The University of Georgia, Athens, Georgia 30602, United States of America

4. Department of Horticultural Science, North Carolina State University, Raleigh, North Carolina 27695, United States of America

5. Department of Entomology, College of Agricultural and Environmental Sciences, The University of Georgia, Athens, GA 30602, USA

*: corresponding author email is cyli@uga.edu

**Ground removal and bush point denoising**

The proposed algorithms successfully removed ground and noise points for various sized bushes (Figure S1). Agronomic practices of blueberry plantation resulted in a special pattern where the elevated bed was higher than the surrounding areas. In particular, the row middles of the experimental field in North Carolina were deep furrowed and showed a clear raised bed on which the plants were established. The ground surface under blueberry bushes was curved which presented difficulties in using traditional planar detection algorithms for ground removal. The proposed adaptive algorithm actually utilized this special ground pattern to identify the height threshold (the connection part) between bush main canes and the ground surface. In a raw bush point cloud, the ground and bush occupied more space than the connection part, resulting in two peaking regions in the point height histogram of that bush point cloud. The peaking region in a lower height value range represented the ground, whereas the peaking region in a higher height value range represented the bush. The connection part was a local minimum between the two peaking regions and close to the ground peaking region. The first criterion (the threshold's bin gradient was near zero) selected all local minima in the histogram. The second criterion (the threshold was greater than the lower limit of the bin with the least gradient value) eliminated local minima that had height values lower than the ground peak. The third criterion (the threshold was as small as possible) ensured the selection of the lowest height value to avoid over-removal of non-ground points.

For noise points, the statistical outlier removal (SOR) filter detected scattered points generated from matching errors in point cloud reconstruction and occasional incompletion of ground removal, whereas the DBSCAN algorithm recognized isolated point clusters caused by small irrelevant objects such as weeds or nonblueberry plant materials present in the field. Obvious noise points were removed to form clean point clouds of blueberry bushes, reducing potential errors for trait extraction.


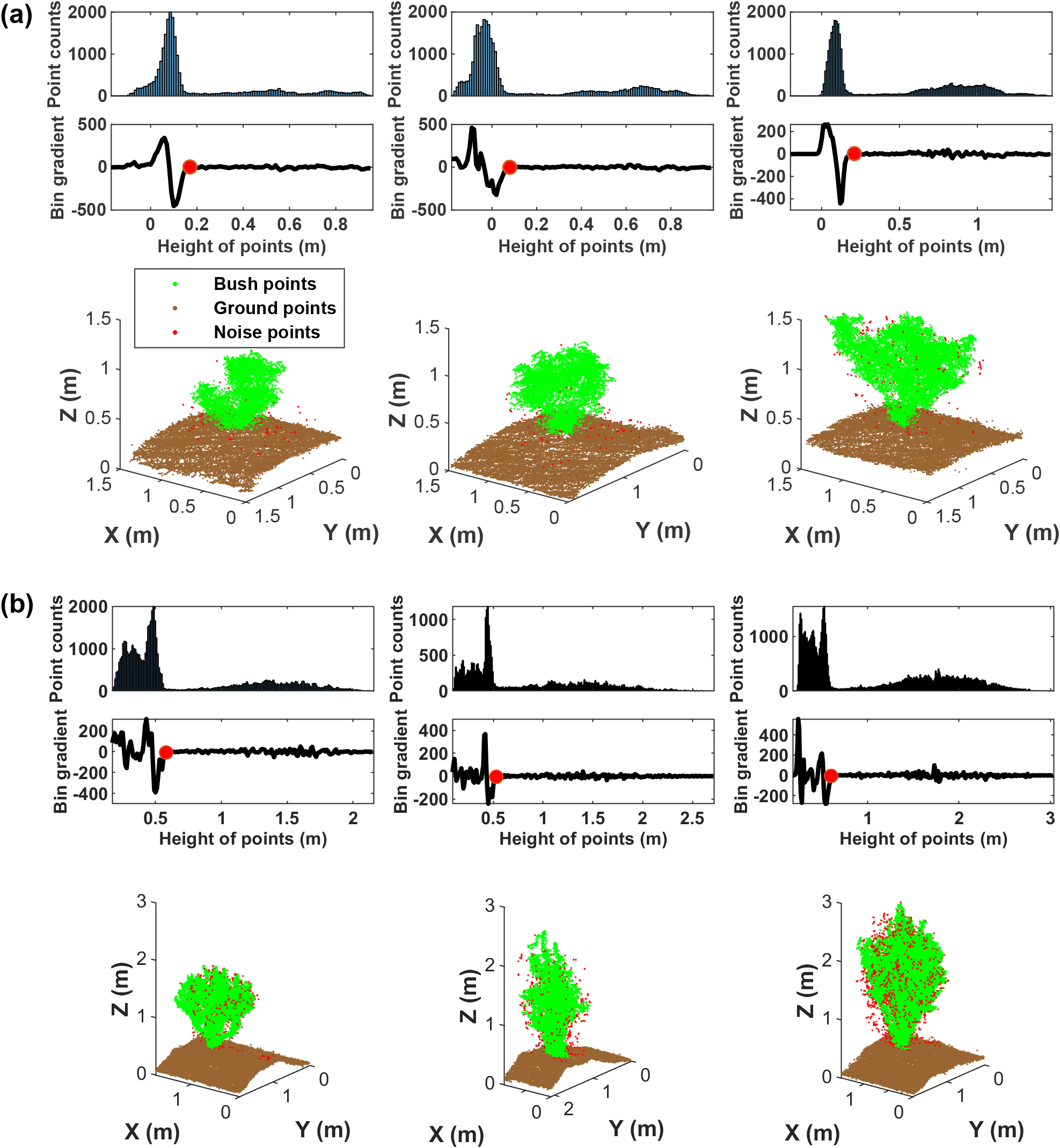


**Figure S1.** Results of representative blueberry bushes for detection and removal of ground and noise points: (a) three bushes in Horticulture Farm of the University of Georgia and (b) three bushes in Horticulture Research Station in North Carolina. The three bushes represented small (left), medium (middle), and large (right) bushes in each experimental field. In each subpanel, the top charts are height histograms, bin gradient curves, and determined thresholds (red dots) of ground points; and the bottom charts are classified point clouds with green color for bush points, brown for the ground, and red for noise.

**Validation of measurement accuracy**

The ZEB1 scanner has a nominal measurement uncertainty of up to 3 cm in all three dimensions. Prior to using the scanner for bush structure quantification, it is necessary to validate the nominal accuracy in field conditions. To avoid effects caused by irregular object shapes, objects with standard shapes were used to test the scanner performance. The objects included five cardboard boxes and one plastic ball. The boxes were directly placed on the ground, whereas the ball was held by a holder on a folding table to maximize the visibility of the ball's bottom hemisphere to the scanner (Figure S2). These objects were scanned in the field with a regular walking pace (approximately 1.4 m/s). Point clouds of individual objects were manually segmented and reoriented to measure geometric parameters including width (x-axis), length (y-axis), and height for boxes and diameters in all three directions for the ball. The width, length, and height (or diameter in each direction) were the distances between lower and upper limits in x, y, z directions, respectively. Volume was calculated using the corresponding convex and concave hulls generated from the point clouds. One-dimensional geometric parameters were manually measured, and volume was calculated based on rectangle or sphere volume formulae for references. Linear regression analyses were performed between sensor and manual measurements, and sensor measurement performance was evaluated using the coefficient of determination (R^2^), root mean squared errors (RMSEs), mean absolute errors (MAEs), and mean relative errors (MREs).


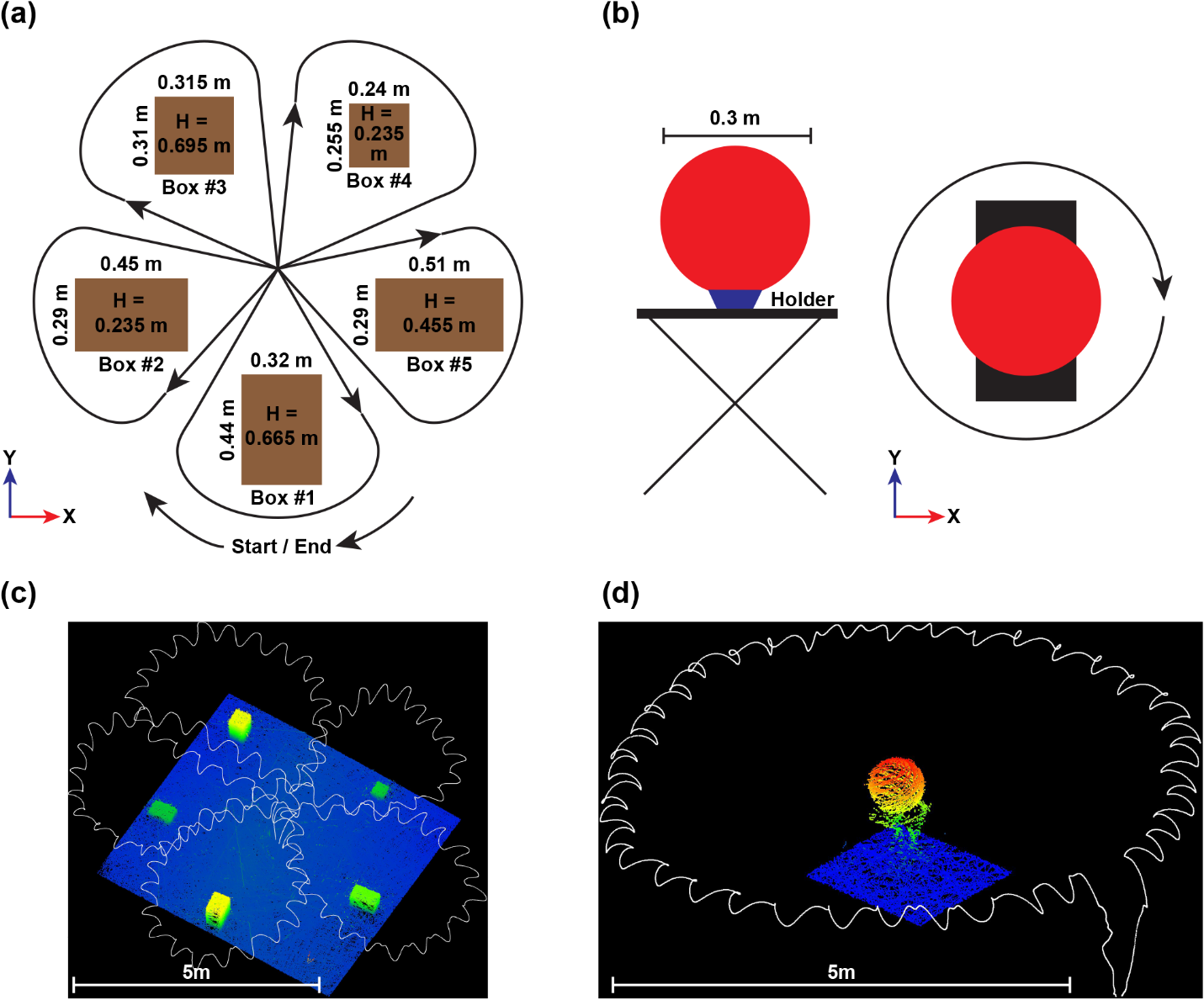


**Figure S2.** Arrangement and raw point clouds of standard-shape objects in the field for validation of the scanner accuracy: (a) and (b) were arrangement of cardboard boxes and plastic ball; (c) and (d) were reconstructed 3D point clouds of cardboard boxes and plastic ball. In (a) and (b), black arrowed lines indicated walking path and direction during field data collection. In (c) and (d), white curves indicated walking patch and oscillation of instrument LiDAR node.

For single dimensional traits (height, width, length), sensor measurements were strongly correlated (R^2^ > 0.96) with manual measurements. RMSEs and MAEs were approximately equal to the scanner nominal accuracy (2-3 cm), indicating the efficacy of using the scanner to accurately measure objects in a certain dimension. For volume, the convex volume measurements had a strong correlation (R^2^ = 0.96) with manual measurements. Although the MRE of convex volume measurements was larger than those of single traits, it was acceptable because the boxes and sphere were in relatively small sizes. Given the nominal accuracy, a small object tended to have a greater relative measurement error than a large object. The correlation and MRE of concave volume measurements deteriorated, because the concave hull method would tightly fit raw point clouds and amplify measurement uncertainties, especially for small objects such as the boxes and ball used in the present study. However, due to this feature, the concave hull method could accurately measure volume for objects with irregular shapes such as blueberry plants. This needs to be further evaluated by comparing sensor and manual measurements of bushes. Nonetheless, standard shapes were more ideal objects than blueberry bushes, because they were not affected by environmental factors (e.g. winds) due to their rigidity. Therefore, the measurement accuracies of standard shapes could be used as the baseline to assess accuracies of measuring blueberry bushes.


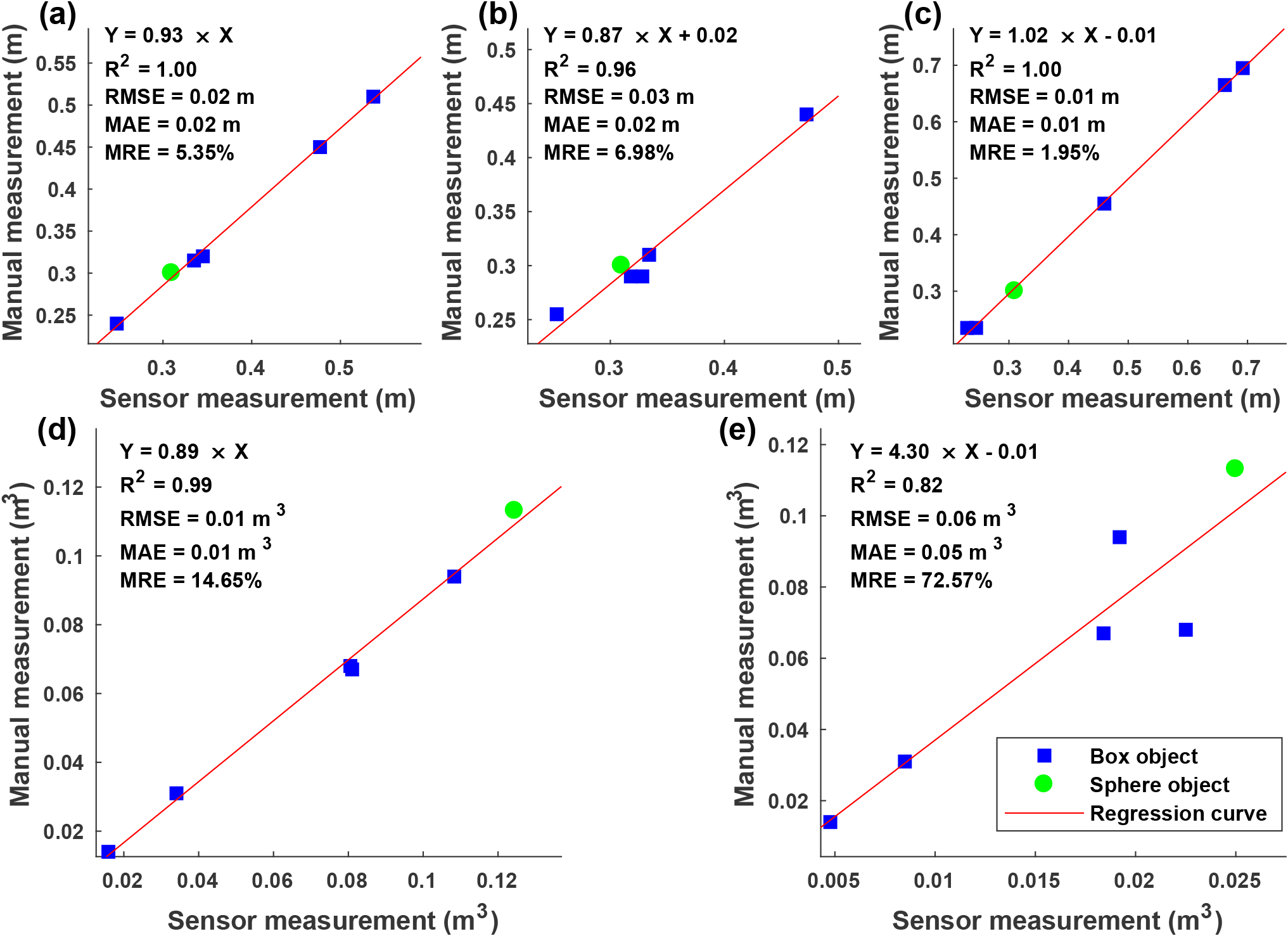


**Figure S3.** Regression results between manual and sensor measurements for standard-shaped objects. (a), (b), (c), (d) and (e) were for height, width (x-axis), length (y-axis), and volume estimated using convex and concave hulls.


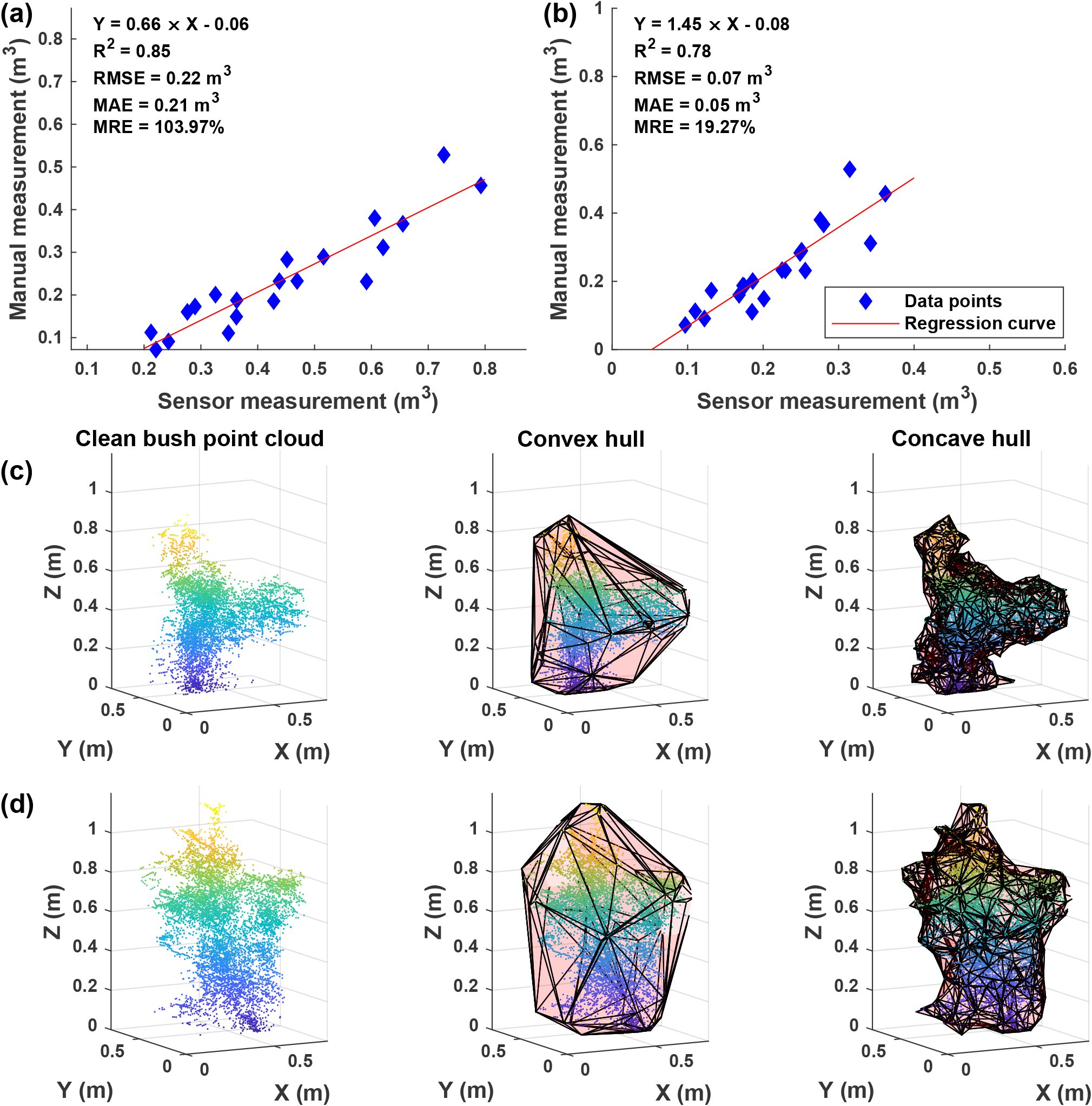


**Figure S4.** Accuracy of bush volume measurements. (a) regression results between bush volume estimated using convex hull and manual methods; (b) regression results between bush volume estimated using concave hull and manual methods; and (c) and (d) illustration of the difference between convex and concave hulls for Bush14 (relatively irregular structure) and Bush19 (relatively regular structure) in Horticulture Farm of the University of Georgia. Manual, convex hull, and concave hull measurements were 0.10, 0.22, and 0.07 m^3^ for Bush14, and 0.2, 0.33, and 0.19 m^3^ for Bush19. Relative differences between convex hull and manual volume measurements were 120% and 65% for Bush14 and Bush19, and relative differences between concave hull and manual volume measurements were 30% and 5% for Bush14 and Bush19.


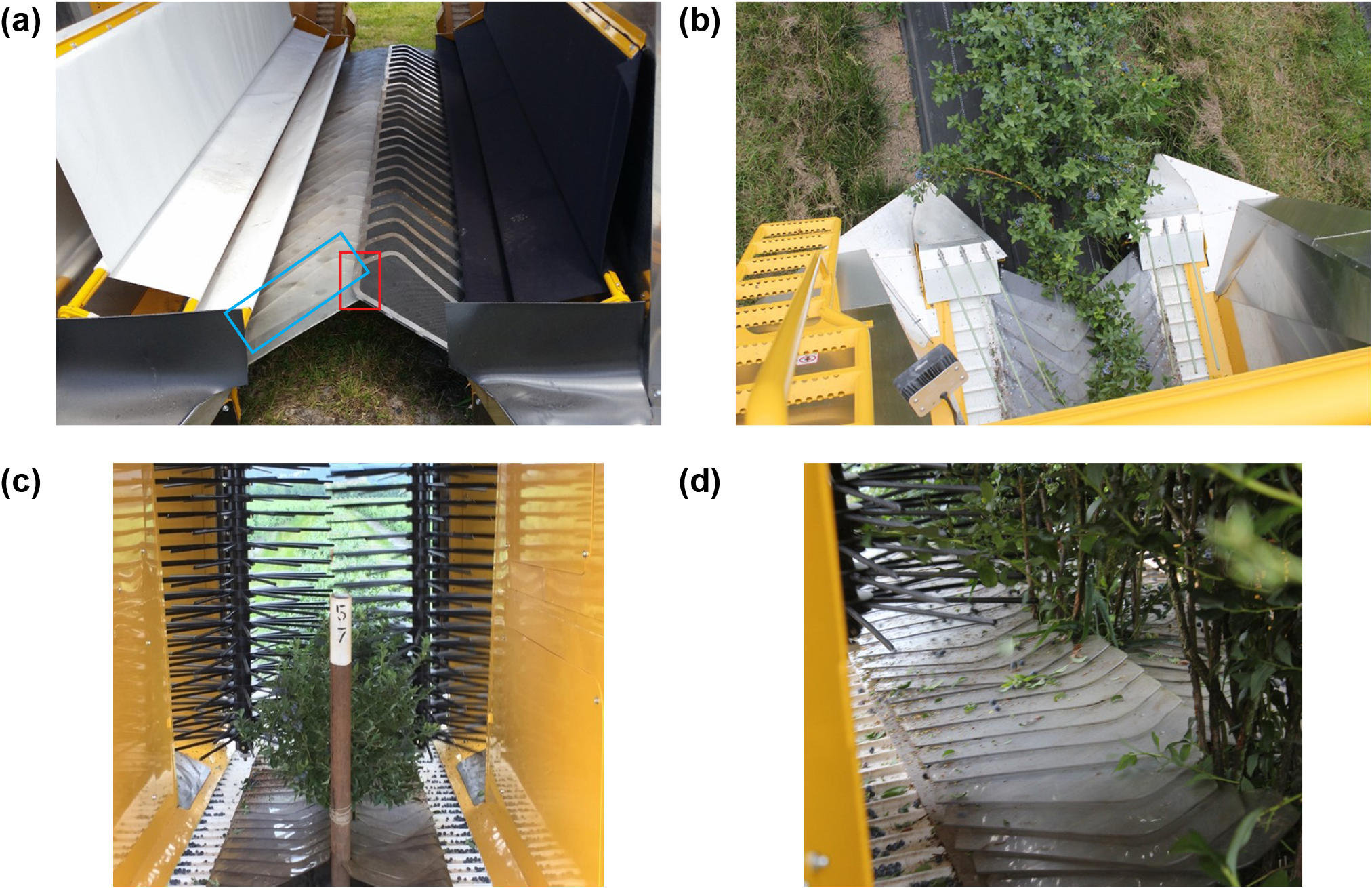


**Figure S5.** Catch plates of over-the-row (OTR) blueberry harvesters: (a) catch plates are installed on either side of the harvester, with large overlaps with plates on the same side (blue region) and a small overlap with plates on the opposite side (red region); (b) top view of catch plates that contact with blueberry plants; (c) inside view of the harvester with a rotary drum shaker with whorls of tines on either of the harvester and catch plates that contact with a single trunk and an endpost; Note the fruit conveyance belt (white ) located outside of catch plates; and (d) a closer view of catch plates that are contacting the crown of blueberry bushes. Note that catch plates contacting the blueberry bush are retracted backward Based on the details provided in (c) and (d), catch plates would fit around a small crown with a smaller gap than around a large crown, reducing the potential of yield loss.


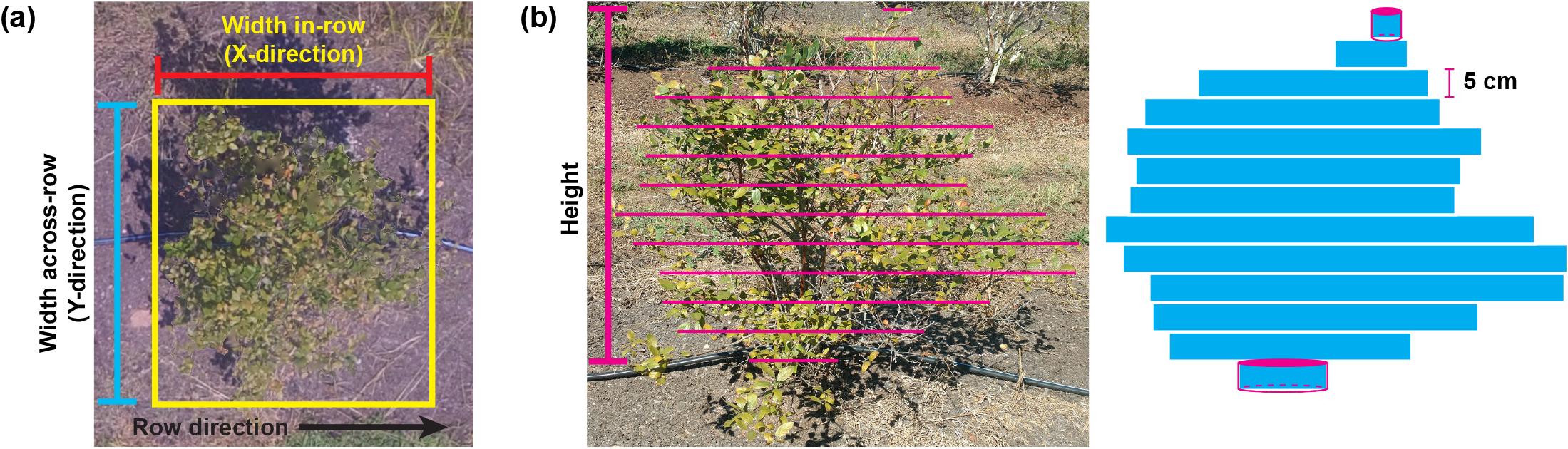


**Figure S6.** Illustration of manual measurement of size traits. (a) Top-view image of a blueberry bush. Row and across-row directions were defined as the x- and y-axes, and the maximum lengths along the two axes were the width in-row and across-row, respectively. Crown size was measured as the bush across-row diameter of the cross-section at 0.15 m above the ground. (b) Bush height was the distance between the highest point of a bush and ground surface. A cylindrical model was used for bush volume measurement. A blueberry bush was visually segregated into layers with an interval of 5 cm in vertical direction. Each layer was considered as a cylinder, with a diameter estimated using the slice perimeter manually measured.
